# The Yeast‐Fermented Garlic and a Balance of Spermine/Spermidine Activates Autophagy via EGR1 Transcriptional Factor

**DOI:** 10.1002/mnfr.202400606

**Published:** 2025-02-13

**Authors:** Kun Xie, Satoshi Yano, Jinyun Wang, Shota Yamakoshi, Tomoe Ohta, Takuhiro Uto, Maiko Sakai, Xi He, Kaichi Yoshizaki, Takumi Kubota, Kohta Ohnishi, Taichi Hara

**Affiliations:** ^1^ Laboratory of Food and Life Science Faculty of Human Sciences Waseda University Tokorozawa Japan; ^2^ College of Animal Science and Technology Hunan Agricultural University Changsha Hunan China; ^3^ Faculty of Pharmaceutical Sciences Department of Pharmacognosy Nagasaki International University Sasebo Nagasaki Japan; ^4^ Department of Clinical Nutrition and Food Management Institute of Biomedical Sciences Tokushima University Graduate School Tokushima Japan; ^5^ Department of Disease Model Institute for Developmental Research Aichi Developmental Disability Center Aichi Japan; ^6^ Tenshindo Co. Ltd Koto‐ku Tokyo Japan

**Keywords:** autophagy, EGR1, garlic, spermidine, spermine, transcriptome analysis

## Abstract

Spermine (SPM) and spermidine (SPD) are polyamines found in all organisms, and their concentrations can be regulated by ingestion. We demonstrated that yeast‐fermented garlic (YF) extract significantly increased autophag flux in OUMS‐36T‐1 and HeLa cells expressing the fluorescent probe (GFP‐LC3‐RFP‐LC3ΔG). YF‐induced increase of autophagy occurred independently of mTORC1 signaling, and RNA‐sequencing analysis revealed that *EGR1* was the most significantly altered gene in YF‐treated OUMS‐36T‐1 cells. YF‐treated *EGR1‐*deficient HAP1 cells displayed reduced autophagic flux (*p *< 0.05). YF‐induced increasing of autophagic flux occurred via a specific SPM/SPD ratio. HAP1 cells treated with equivalent amounts of SPD or SPM as that found in YF did not increase autophagic flux (*p* > 0.05); however, treatment with SPD and SPM in the same ratio as that found in YF increased autophagic flux (*p* < 0.05). This specific SPM/SPD ratio reduced MG132‐induced proteostress via *EGR1*‐dependent pathways (*p* < 0.05). Thus, the SPM/SPD balance may regulate autophagy via *EGR1*‐dependent pathways, and controlling this balance may provide a strategy to maintain cellular homeostasis.

## Graphical abstract

Spermine (SPM) and spermidine (SPD) are polyamines crucial for cellular functions, and their balance can be influenced by dietary intake. Yeast‐fermented garlic (YF) extract was shown to significantly enhance autophagic flux in vitro, independently of mTORC1 signaling. RNA sequencing revealed that EGR1 plays a critical role in mediating YF‐induced autophagy. The specific SPM/SPD ratio present in YF was identified as essential for this effect, as neither SPM nor SPD alone replicated the autophagy‐enhancing activity. This ratio also alleviated proteostress through EGR1‐dependent pathways, highlighting the importance of SPM/SPD balance in regulating autophagy and maintaining cellular homeostasis.

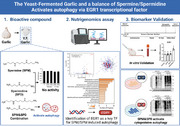

## 1 Introduction

As the world population ages, chronic diseases such as diabetes, cancer, and neurodegeneration become increasingly prevalent. Interventions that favor healthy aging should constitute powerful strategies to limit human diseases with broad socioeconomic impacts. Intermittent fasting or caloric restriction, which activate autophagy, extends the healthy lifespan of all tested model organisms [1]. Conceptually, healthy aging requires the retardation of multiple molecular and cellular alterations, including genomic instability, loss of protein degradation capacity, mitochondrial dysfunction, cellular senescence, and chronic inflammation, which drive the aging process and induce age‐associated pathologies [2].

Autophagy is a cellular process involving the lysosomal degradation of cytoplasmic components, enabling the recycling of cytoplasmic materials, and eliminating damaged or dysfunctional cell constituents. Autophagy has been implicated in many physiological and pathological settings, including human diseases [3]. Accordingly, accurate quantification of the overall process of autophagic degradation, termed “autophagic flux,” is crucial in autophagy studies. Furthermore, recent studies have shown that autophagy is involved in determining the life span of many model organisms [4]. For instance, reduced autophagy has been associated with accelerated aging [4, 5], whereas stimulation of autophagy may facilitate potent anti‐aging effects and prevent the development of aging‐associated pathologies [5]. At the molecular level, autophagy initiation is canonically activated by the inhibition of mechanistic target of rapamycin (mTOR), which unleashes a cascade of down‐stream target dephosphorylation, such as p70 S6 kinase (p70 S6K) and eukaryotic translation initiation factor 4E‐binding protein 1 (4EBP1), to generate a primordial membrane, known as the phagophore, that extends by acquiring additional lipids to form the mature double‐membrane autophagosome [6]. The amounts of autophagosomes are marked by the phosphatidylethanolamine (PE)‐conjugated form of MAP1LC3/LC3 (microtubule‐associated protein 1 light chain 3; termed LC3‐II), a homolog of yeast Atg8 (autophagy‐related 8) [7]. Therefore, we used  the fluorescence probe of GFP‐LC3‐RFP‐LC3ΔG (or RFP) to monitor autophagic flux in which the GFP fluorescence is lost when the GFP‐LC3 is delivered to the lysosome for degradation, whereas RFP‐LC3ΔG (or RFP) remains stable and continues to emit red fluorescence. Therefore, the autophagic flux can be accurately assessed by measuring the ratio of GFP to RFP fluorescence by fluorescence microscopy or fluorescence‐activated cell sorting (FACS) [8].

**FIGURE 1:**
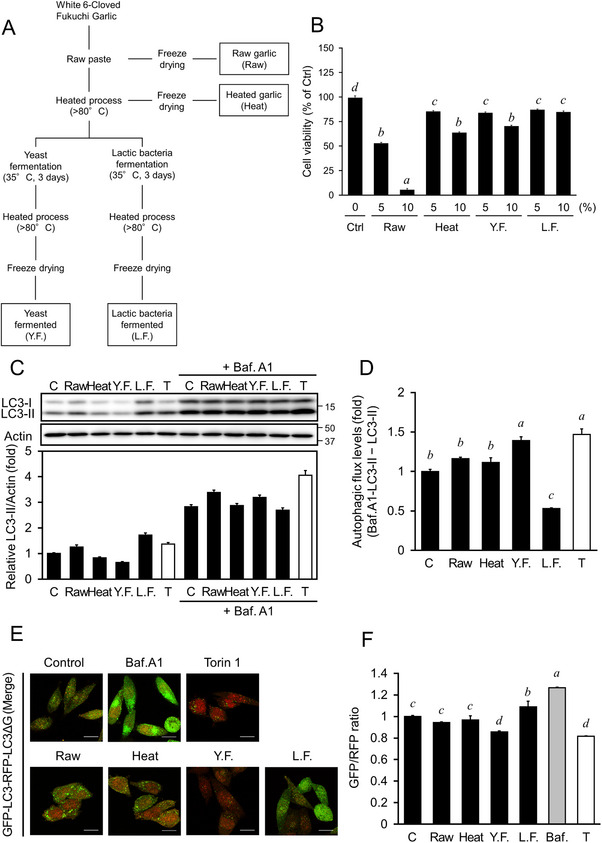
An evaluation of autophagic flux in cells treated with novel processed garlic foods. (A) A scheme indicating the garlic processing procedure. (B) OUMS‐36T‐1 cells were treated with 5%–10% water extracts of raw garlic (Raw), heated garlic (Heat), yeast‐fermented garlic (YF), or *Lactobacillus*‐fermented garlic (LF) for 24 h. The cell viability rate was assessed using MTT assay and was expressed as the optical density ratio of the treatment to control. (C) LC3 protein levels were measured after OUMS‐36T‐1 cells were treated with 5% water extracts of Raw, Heat, YF or LF, or 1 µM Torin‐1 (T), in the presence or absence of 200 nM Bafilomycin A1, a vacuolar H+‐ATPase inhibitor (Baf. A1), for 4 h. The whole cell lysate was used for western blot analysis with the indicated specific antibodies. Densitometry of the blots was performed using the FUSION SOLO S (Vilber Lourmat). (D) Calculations for net LC3‐II flux levels in densitometric data of (A). The level of LC3‐II in each sample was normalized to the level of actin, and the amount of autophagic flux was calculated by subtracting the amount of LC3‐II in the Baf. A1‐treated sample (inhibited lysosomal degradation) from the amount of LC3‐II in the Baf. A1‐untreated sample (normal lysosomal degradation). The values for the untreated sample (Ctrl) were set to 1, and the values for the other samples were expressed relative to Ctrl. (E) HeLa cells expressing GFP‐LC3‐RFP‐LC3ΔG were treated with 5% Raw, Heat, YF or LF, 1 µM T, or 200 nM of Baf. A1 for 4 h, followed by fixation using 2% paraformaldehyde. Fluorescence was visualized using confocal laser scanning microscopy (FV3000, Olympus). The bar indicates 20 µm. (F) Quantitation of autophagic flux. Cell treatments were the same as in (C). Cellular fluorescence intensity was measured using Cellometer Vision (Nexcelom Bioscience LLC), and FCS Express4 (De Novo software) was used for quantitative analysis. The data represent the mean ± SEM (*n* = 3). Different letters indicate varying levels of significance (*p* < 0.05) among the groups analyzed with Tukey's test after one‐way ANOVA. ANOVA, analysis of variance; SEM, standard error of the mean.

Garlic (*Allium sativum*), a well‐known plant, is used in dietary strategies for treating various ailments and as an antibiotic, an anti‐thrombotic, and an anti‐neoplastic agent [9]. The bioactive components extracted from garlic exhibit promising therapeutic potential to induce mitophagy and to treat metabolic disorders [10]. Agro‐industrial and other biotechnological processing techniques are widely employed to increase the efficacy and bioavailability of garlic; however, processing significantly disrupts the activity of bioactive compounds found in garlic [11, 12]. For instance, when the garlic bulb is damaged, the vacuolar enzyme alliinase in garlic rapidly lyses the cytosolic cysteine sulfoxides (alliin) to allicin, which is unstable and decomposes within 16 hours [13]. Fukuchi White 6 Clove, a representative garlic breed from Aomori, Japan, is the standard garlic cultivar and has the largest areal distribution in Japan. Commercial products of this garlic include raw, heated, *Saccharomyces cerevisiae* (yeast)‐fermented, and *Lactobacillus*‐fermented garlic (Raw, Heat, YF, and LF, respectively; Figure 1). The alteration in bioactive components of garlic after processing and their effects on human health remain unclear.

Dietary polyamine concentration in garlic increases during fermentation [12]. Polyamines are essential polycations present in all living organisms. They participate in the regulation of several important cellular processes, such as gene transcription and translation, cell growth, and proliferation. Polyamines are believed to exert their effects by modulating protein–protein and protein–DNA interactions. Cellular polyamine levels decline with aging and can only be regulated via dietary uptake and de novo synthesis [16]. Polyamine deficiency is associated with various pathologies, such as neurological abnormalities, malignancies, and aging [14, 15]. Elevated dietary polyamines show several effects in humans, including anti‐inflammatory and anti‐oxidant properties, enhanced mitochondrial metabolic function and respiration, and activation of cytoprotective autophagy, though the mechanisms are not fully understood [16, 17, 18]. Thus, this study aimed to define the bioactive components of garlic after yeast fermentation and examine their effect on autophagy.

## 2 Materials and Methods

### 2.1 Preparation of Samples and Reagents

Processed Fukuchi White 6 Clove garlic was purchased from Miyagi, Japan. Spermidine (SPD) and spermine (SPM) were purchased from Wako, Fujifilm (Tokyo, Japan). Polyamine‐TAMRA/DAPI staining reagents were purchased from Funakoshi (Tokyo, Japan), and MitoSOX Red reagent was purchased from Thermo Fisher Scientific (Waltham, MA, USA). Molecular weight cutoff ultrafiltration membranes were purchased from Sigma–Aldrich (Millipore‐Sigma, Darmstadt, Germany). Antibodies against phosphorylated(p)‐70 S6K, translation repressor protein 4EBP1, ubiquitinated proteins, and β‐actin were purchased from Cell Signaling Technology (CST, Tokyo, Japan). The antibody against Early Growth Response 1 (*EGR1*) was purchased from Sigma–Aldrich (Millipore‐Sigma, Darmstadt, Germany). MG132 was purchased from the Peptide Institute, Japan. Protease inhibitor cocktail (complete EDTA‐free protease inhibitor) was purchased from Roche (Indianapolis, IN, USA). Bafilomycin A1 and Torin 1 were purchased from Cayman Chemical (Ann Arbor, MI, USA).

### 2.2 Extraction of Novel Processed Garlic Foods

White 6 clove fukuchi garlic was used to make a paste; this paste was termed raw garlic (Raw). Raw was heated to 80°C or higher for sterilization and termed heated garlic (Heat). Heat was fermented by *S. cerevisiae* or Lactobacilli with glucose at 35°C for 3 days, followed by heating at 80°C or higher, respectively, and was termed yeast‐fermented garlic (YF) and lactic bacteria‐fermented garlic (LF), respectively. All processed garlic compounds were freeze‐dried without excipients. These processed garlic compounds were extracted in water (1 g/5 mL) at 4°C for 1 day, and then were centrifuged at 10000 × *g* for 10 min. The supernatants were filtered (0.22 µm) before the experiments.

### 2.3 Cell Culture

OUMS‐36T‐1 cells, an *hTRT* gene transfected normal human embryo fibroblast cell line, were obtained from the Japanese Collection of Research Bioresources (Tokyo, Japan). HeLa cells, a human cervix epithelioid carcinoma cell line, were obtained from the American Type Culture Collection (Manassas, VA, USA). Both cells were cultured in Dulbecco's Modified Eagle Medium (Gibco, New York, USA) supplemented with 10% fetal bovine serum and 1% penicillin–streptomycin (Fujifilm) at 37°C in a humidified 5% CO_2_ atmosphere. HAP1 cells and *EGR1*‐deficient HAP1 cell lines were obtained from Horizon Discovery (Cambridge, UK). HAP1 cells were cultured in Iscove's Modified Dulbecco's Medium (IMDM) supplemented with 10% fetal calf serum and penicillin/streptomycin (Wako) in a 5% CO_2_ incubator at 37°C.

### 2.4 Administration Details

Pharmacological analysis in vitro to investigate the potential toxicity of the garlic extracts was conducted via cell viability assay. The in vivo application of SPM and SPD was regarding the in vitro analysis and previous reports [19].

### 2.5 Animal Management

The animal experimental protocol was performed according to the guidelines of the Animal Care and Use Committee of Hunan Agricultural University (Permission No. A12005). The C57BL/6N 8‐week‐old male mice purchased from SLC Inc. (Cyagen Biosciences, Suzhou, China) were housed separately in cages with wood shaving bedding, controlled light (12 h/day) and temperature (25°C). After a 1‐week acclimatization period, the mice were randomly divided into four treatment groups: control, SPM, SPD, and SPM/SPD. A 34 mM SPD solution, a 10 mM SPM solution, and a mixed solution containing 34 mM SPD and 10 mM SPM (at the same ratio as used in the in vitro experiments) were prepared. Following an overnight fast, the mice were re‐fed with standard chow the next morning to prevent starvation‐induced autophagy. Four hours after refeeding, the mice received intragastric administration of the respective treatments: the control group received 100 µL of water, the SPD group received 100 µL of the SPD solution, the SPM group received 100 µL of the SPM solution, and the SPM/SPD group received 100 µL of the mixed SPD and SPM solution.

Blood was collected from the eyeball, the RNA from blood samples was isolated using the Mouse RiboPure‐Blood RNA Isolation Kit (Thermo, Waltham, MA, USA). Immediately afterward, reverse transcription was performed with the PrimeScript II 1st Strand cDNA Synthesis Kit (Takara, Tokyo, Japan). The resulting cDNA was subsequently used for qPCR analysis.

### 2.6 Generation of Transgenic Cells Expressing GFP‐LC3‐RFP‐LC3ΔG or GFP‐LC3‐RFP

HeLa cells were transfected with pMRX‐IP‐GFP‐LC3‐RFP‐LC3ΔG (Addgene plasmid #84572; http://n2t.net/addgene:84572; RRID: Addgene_84572) using lipofectamine 2000, and puromycin‐resistant cells were selectively cultured for 2 weeks. Thereafter, the cells were subjected to single‐cell sorting using a JSAN cell sorter (Bay Bioscience, Hyogo, Japan) to yield a single clone that strongly emitted both GFP and RFP. HAP1 cells expressing GFP‐LC3‐RFP were generated via retroviral transduction. To generate retroviruses, Plat‐E cells were cotransfected with pMRX‐IP‐GFP‐LC3‐RFP using FuGENE HD (Promega). HAP1 cells were then infected with the recombinant retroviruses and selected in a medium containing 10 µg/mL puromycin.

### 2.7 Cell Viability Assay

The cell viability was measured MTT assay (Dojindo, Tokyo, Japan). OUMS‐36T‐1 cells (5.0 × 10^3^/well) were seeded in 96‐well plates and treated with the indicated concentrations of Raw, Heat, YF, and LF water extracts for 24 h. Ten microliters MTT solution (5 mg/mL) was added to each well and incubated for another 4 h. The amount of purple formazan dye was determined by measuring the absorbance at 570 nm using a Multiskan FC (Thermo, Waltham, MA, USA). The cell proliferation rate was expressed as the optical density ratio of the treatment to control.

### 2.8 Measurement of Mitochondrial Superoxide Levels

The mitochondrial superoxide levels were measured by MitoSOX Red (Invitrogen). Both HeLa cells and HAP1 cells were pretreated with 5% YF or SPM/SPD water extracts (1.0 µM/3.4 µM) for 12 h, followed by exposure to 10 µM MG132 for 12 h and staining with 10 µM of MitoSOX RED for 1 h. The cells were collected, and the fluorescence intensity was measured using Cellometer Vision (Nexcelom Bioscience LLC). Next, FCS Express4 (De Novo software) was used for quantitative analysis.

### 2.9 Quantitative Real‐Time PCR

OUMS‐36T‐1 cells were seeded in a 6‐well plate (1.0 × 10^5^/well), followed by treatment with garlic water extracts for 4 h. Total RNA was extracted using ReliaPrep RNA Cell Miniprep Systems (Promega Co., Ltd. Madison, USA), following the manufacturer's instructions. Reverse transcription was performed using ReverTra Ace qPCR RT Master Mix with gDNA Remover (Toyobo Bio, Inc., Tokyo, Japan) according to the manufacturer's manual. Briefly, after incubating at 65°C for 5 min, extracted RNA was reacted with 4 × sDN Master Mix at 37°C for 5 min, after which reverse transcription was performed using 5 × RT Master Mix II at 37°C for 15 min, followed by 98°C for 5 min. qPCR was performed using a Thermal Cycler Dice Real Time System III (Takara Bio, Inc., Tokyo, Japan) with TB Green Premix Ex Taq II (Tli RNaseH Plus; Takara Bio, Inc.). The thermal cycling condition was held at 95°C for 30 s, followed by 40 cycles of 5 s at 95°C and 30 s at 60°C. Primers used are listed in Table S1. The relative mRNA expression levels were calculated using the ΔΔC_t_ method and normalized against *GAPDH* as the internal control.

### 2.10 Western Blotting

The cells were lysed with Tris‐Triton buffer containing 50 mM Tris‐HCl (pH 7.4), 150 mM NaCl, 1 mM EDTA, 1% TritonX‐100, and a proteinase inhibitor cocktail. The cell lysates were centrifuged at 15 000 × *g* for 15 min and the supernatants were collected. The protein concentration was determined using a Protein Assay bicinchoninic acid Kit (Fujifilm). Equal amounts of lysate protein were separated via sodium dodecyl‐sulfate polyacrylamide gel electrophoresis and transferred to a polyvinylidene difluoride membrane (Merck KGaA, Darmstadt, Germany). The membrane was first blocked with Tris‐buffered saline with 0.1% Tween 20 Detergent buffer (500 mM NaCl, 20 mM Tris‐HCl [pH 7.4], and 0.1% Tween 20) containing 5% nonfat dry milk, and then incubated with specific antibodies overnight at 4°C, followed by horseradish peroxidase‐conjugated secondary antibodies for another 1 h after the overnight incubation. Bound antibodies were detected using the electrochemiluminescence system, and the relative amounts of proteins associated with specific antibodies were quantified using FUSION SOLO S (Vilber Lourmat, Marne‐la‐Vallée, France).

### 2.11 Confocal Laser Scanning Microscopy

After treatment with garlic water extracts for 4 h, HeLa cells expressing GFP‐LC3‐RFP‐LC3ΔG were fixed in 2% paraformaldehyde, followed by mounting with SlowFade Diamond Antifade Mountant (Invitrogen). Cellular fluorescence was visualized using confocal laser scanning microscopy (FV3000, Olympus).

### 2.12 Determination of Cellular Autophagic Flux

The cells expressing GFP‐LC3‐RFP‐LC3ΔG (or RFP) were treated with indicated samples or reagents and cultured in 12‐well plates with 1.0 × 10^5^ cells /well for 4h. The cells were washed with PBS and incubated with 0.25 % Trypsin for 2 min. After incubation, cells were collected in 1mL PBS. For autophagic flux assay, the fulorescent intensity of the cells were measured using cellometer vision (Nexcelom Bioscience LLC) or SA3800 Spectral Analyzer (Sony Biotechnology, Tokyo, Japan). Fluorescence intensity of GFP and RFP were quantitated by FCS Express4 (De Novo software), and GFP/RFP ratio was calculated as an index of cellular autophagic flux.

### 2.13 Transcriptional Profiling via RNA Sequencing

OUMS‐36T‐1 cells were treated with YF for 4 h, after which total RNA was isolated using TRIzol Reagent (Invitrogen) according to the manufacturer's protocol. A cDNA library was then constructed using an NEB Next Ultra RNA Library Prep kit for Illumina (New England Biolabs, Inc.) following the manufacturer's protocols. RNA sequencing was performed using a NovaSeq 6000 System by Novogene (Beijing, China). The reads were mapped to the reference sequences using TopHat 2. Genes with an adjusted *q* value < 10^−10^ were identified as differentially expressed genes.

### 2.14 Measurement of Intracellular Proteostasis

Proteasome inhibition leads to protein aggregation in the cytosol and triggers cytotoxic unfolded protein responses. To induce cytotoxic protein aggregation, the cell‐permeable proteasome inhibitor MG132 was used. Upon proteasome inhibition, the elimination of unfolded proteins becomes reliant on autophagy activation. Cells were pretreated with 5% YF or SPM/SPD (1.0 µM/3.4 µM) water extracts for 12 h, followed by treatment with 10 µM MG132 for an additional 12 h. After treatment, the cells were lysed, and equal amounts of protein from the lysates were subjected to western blotting using an anti‐ubiquitin antibody. Following overnight incubation, horseradish peroxidase‐conjugated secondary antibodies were applied for 1 h. The bound antibodies were detected using an electrochemiluminescence system, and the relative protein levels were quantified using the FUSION SOLO S system (Vilber Lourmat, Marne‐la‐Vallée, France).

### 2.15 Statistical Analysis

The data represent the mean ± SEM (*n* = 3). Statistical analysis was performed using one‐ or two‐way analysis of variance (ANOVA), followed by Dunnett's or Tukey's test as a multiple comparison test, or two‐tailed Student's *t* test corrected with the Bonferroni method. A probability of *p *< 0.05 was considered significant.

## 3 Results

### 3.1 Effect of Various Garlic Extracts on Cell Viability and Autophagy

The garlic extracts were prepared accordingly (Figure 1A), and detailed information was described in Supporting Information Data. We first tested cell viability after treatment with different garlic extracts. OUMS‐36T‐1 cells were treated with 5%–10% Raw, Heat, YF, or LF water extracts for 24 h. The viability measured by MTT assay of cells treated with 10% Raw, Heat, YF, or LF decreased significantly (Figure 1B, *p *< 0.05), compared with 10% extracts treatment, 5% garlic extracts had higher cell viability (*p* < 0.05), meanwhile, cell viability measured by CCK‐8 or cell counting assay is shown in Figure S1, the results also showed 5% garlic extracts had higher cell viability (*p* < 0.05). Thus, the 5% garlic extracts were further used to evaluate autophagic flux, and Torin‐1—a mTOR inhibitor—was used as a positive control. Results revealed that YF significantly elevated LC3‐II protein levels with Baf. A1 treatment relative to the amount without treatment (*p *< 0.05), whereas LC3‐II levels in LF‐treated  cells were not significantly altered by Baf. A1 treatment  (Figure 1C, D). The findings suggested that YF increased autophagic flux in OUMS‐36T‐1 cells, whereas LF inhibited cellular autophagy, resulting in LC3 protein accumulation. Furthermore, HeLa cells expressing GFP‐LC3‐RFP‐LC3ΔG were treated with different garlic extracts, and the autophagic flux was evaluated. Consistently, YF‐treated cells displayed relatively lower GFP/RFP ratios than controls (Figure 1E, F, *p *< 0.05), indicating that YF significantly increased autophagy activity.

### 3.2 YF‐induced Autophagy Independent of mTOR Signaling

**FIGURE 2:**
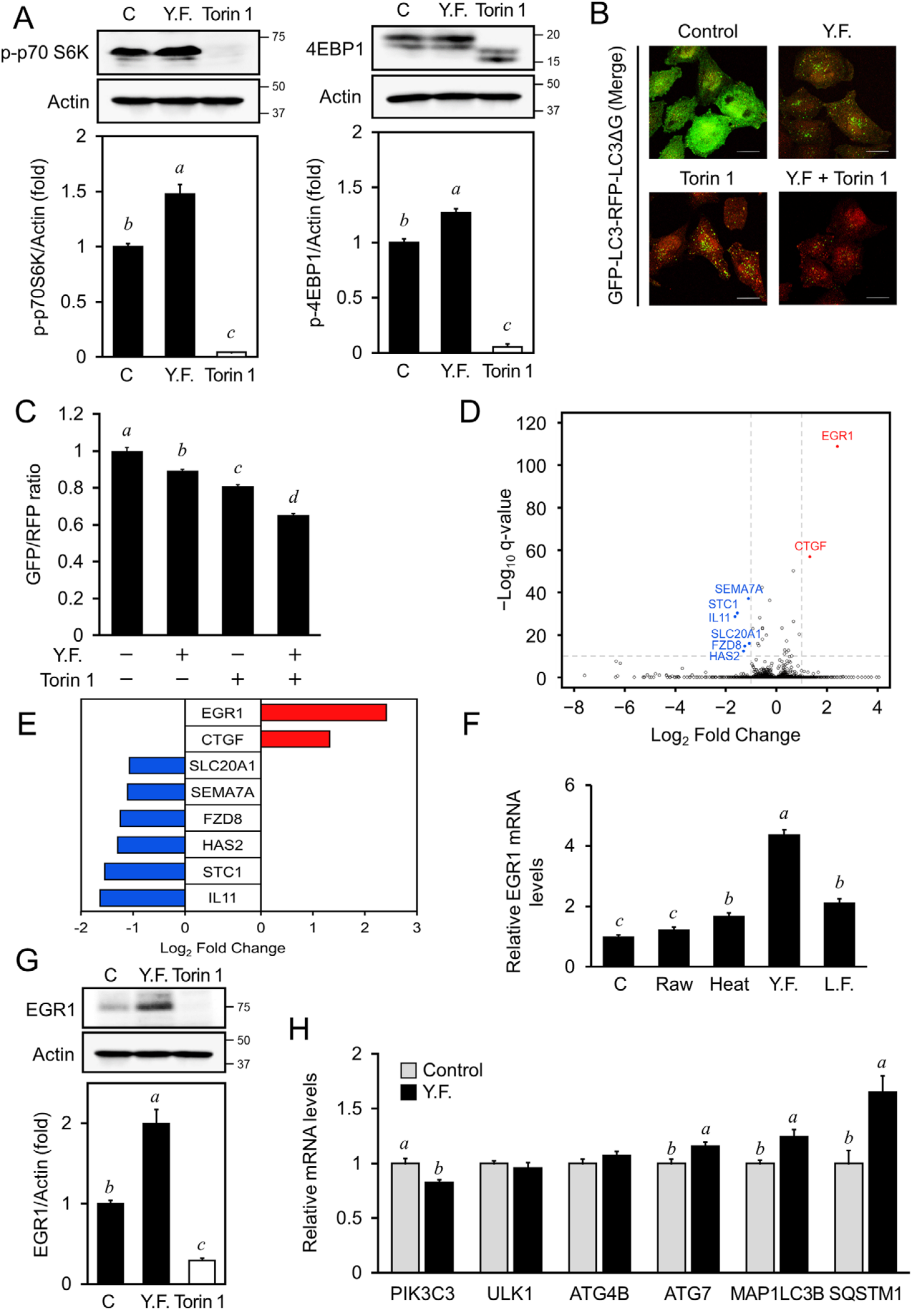
Identification of potential target gene of mTORC1‐independent autophagy in cells treated with yeast‐fermented garlic (YF). (A) The levels of phospho‐p70 S6K and 4EBP1 band shifts. OUMS‐36T‐1 cells were treated with 5% water extracts of YF or 1 µM Torin‐1 (T) for 4 h. Whole cell lysate was used for western blot analysis with specific antibodies. Densitometry was performed using the FUSION SOLO S (Vilber Lourmat). (B, C) Determination of autophagic flux in combination with YF and Torin‐1. (B) HeLa cells expressing GFP‐LC3‐RFP‐LC3ΔG were treated with 5% YF and/or 1 µM T for 4 h, followed by fixation using 2% paraformaldehyde. Fluorescence was visualized using confocal laser scanning microscopy (FV3000, Olympus). The bar indicates 20 µm. (C) Quantitation of autophagic flux. Cell treatments were the same as in (B). Cellular fluorescence intensity was measured using Cellometer Vision (Nexcelom Bioscience LLC), and FCS Express4 (De Novo software) was used for quantitative analysis. (D) Volcano plot of the transcriptional profile of OUMS‐36T‐1 cells treated with 5% water extracts of YF for 4 h. (E) Bar chart showing differentially expressed genes in analysis of (A). (F) Validation of *EGR1* gene expression through qPCR. OUMS‐36T‐1 cells were treated with 5% water extracts of raw garlic (Raw), heated garlic (Heat), YF, or *Lactobacillus*‐fermented garlic (LF) for 4 h. Total RNA was isolated using ReliaPrep RNA Cell Miniprep System, and reverse transcription was performed as described in the manufacturer's manual. The samples were analyzed using real‐time PCR (ΔΔCt method). (G) Quantification of *EGR1* protein levels. OUMS‐36T‐1 cells were treated with YF or 1 µM Torin‐1 for 4 h. Whole cell lysate was used for western blot analysis with specific antibodies. Densitometry of the blots was performed using the FUSION SOLO S (Vilber Lourmat). (H) *EGR1* target gene expression. Cell treatments were the same as in (A) and total RNA isolation and real‐time PCR were the same as in (F). The data represent the mean ± SEM (*n* = 3). The letters indicate varying levels of significance (*p* < 0.05) analyzed with Tukey's test among the groups after one‐way ANOVA. ANOVA, analysis of variance; SEM, standard error of the mean.

mTOR signaling is the canonical pathway regulating autophagy in response to nutrition and growth factor stimuli [20]. Phosphorylation levels of p70 S6K and 4EBP1 are directly regulated by mTORC1 signaling. Activation of mTORC1 leads to phosphorylation of these proteins, which subsequently regulates cell growth. Conversely, when mTORC1 signaling is inhibited, phosphorylation of p70 S6K significantly decreases, and a band shift in 4EBP1 can be observed via western blotting due to changes in phosphorylation levels. Therefore, we measured the phosphorylation levels of p70 S6K and 4EBP1 in cells treated with YF. Phospho‐p70 S6K and 4EBP1 remained unchanged after YF treatment, but Torin‐1 significantly reduced phospho‐p70 S6K and 4EBP1 (Figure 2A, *p* < 0.05). Moreover, cells treated with 5% YF and 1 µM Torin‐1 showed relatively lower GFP/RFP ratios than cells treated with Torin‐1 only (Figure 2B, C, *p *< 0.05), suggesting that YF‐induced autophagy occurs independently of mTOR signaling. To identify the YF‐induced autophagy pathway, RNA was extracted from cells treated with or without 5% YF extract, and RNA‐sequencing was performed to examine any significantly altered genes. *EGR1*, *CTGF*, *SLC20A1*, *SEMA7A*, *FZD8*, *HAS2*, *STC1*, and *IL11* were significantly altered after YF treatment (*p *< 0.05; Figure 2D). Among them, *EGR1* and *CTGF* were significantly upregulated (Figure 2E, *p *< 0.05), whereas *SLC20A1*, *SEMA7A*, *FZD8*, *HAS2*, *STC1*, and *IL11* were significantly downregulated (*p *< 0.05). Additionally, the transcription factor, *EGR1*, was the most significantly upregulated gene (*p *< 0.001, Figure 2D, Figure S2G). Gene Ontology, Kyoto Encyclopedia of Genes and Genomes, and Reactome Pathways Enrichment Analysis based on differentially expressed genes (DEGs) are presented in Figure S2B–D. Protein–protein interaction analysis indicated that *PIK3C3* was the most significantly factor involved in *EGR1* regulation (Figure S2E). *EGR1* mRNA expression and protein levels were significantly increased in YF‐treated cells (Figure 2F, G, *p *< 0.05). The expression levels of *EGR1* downstream target genes were analyzed, and results showed that *PIK3C3* expression was significantly decreased (Figure 2H, *p* < 0.05), while *ATG7*, *MAP1LC3B*, and *SQSTM1* were significantly increased (*p *< 0.05).

### 3.3 YF Treatment Induced EGR1‐Mediated Autophagy Activation

**FIGURE 3:**
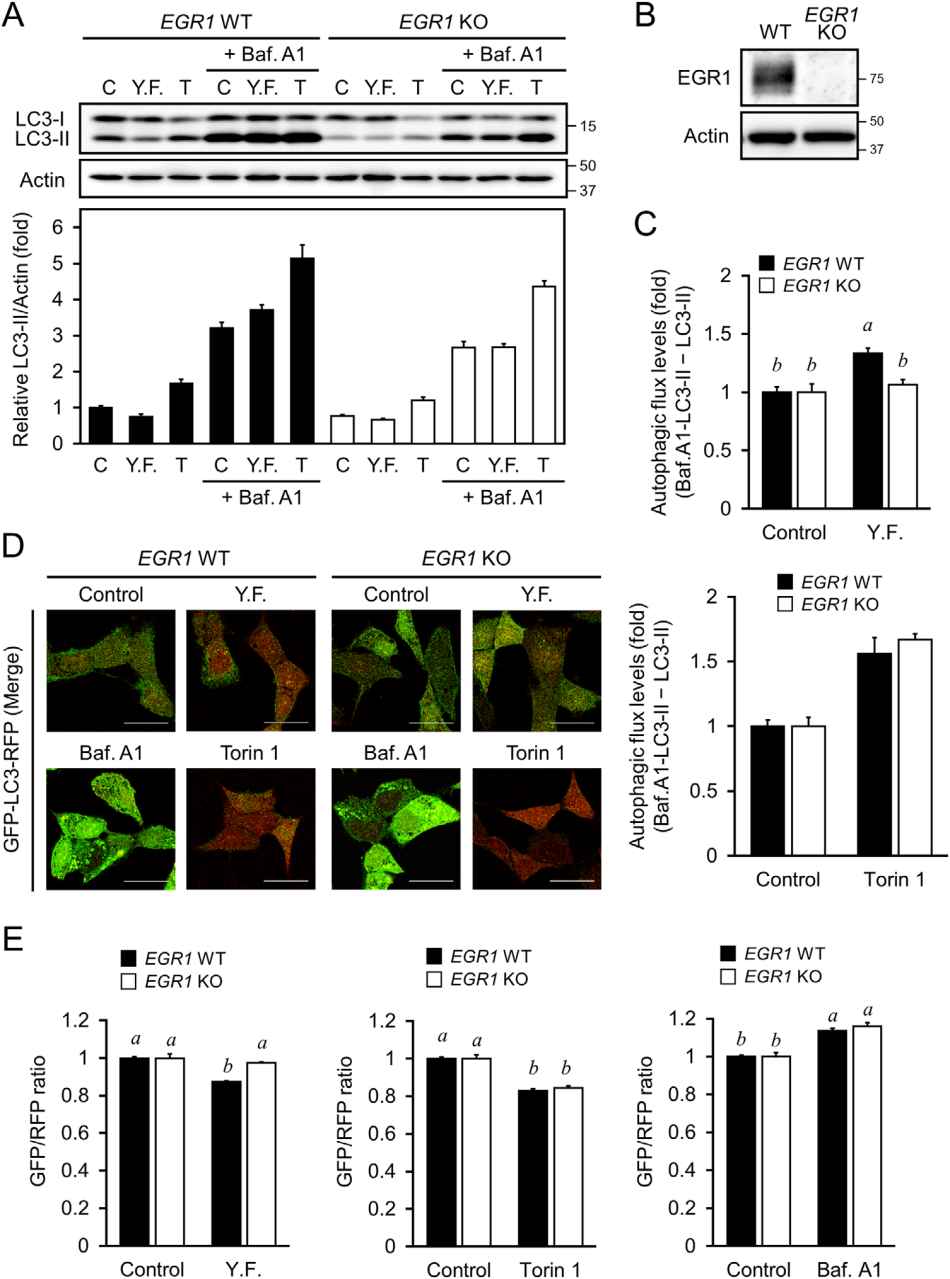
*EGR1*, the novel autophagy‐related gene, functions independently to mTORC1 signaling. (A) LC3 protein levels. HAP1‐WT and HAP1 *EGR1*‐deficient (KO) cells were treated with 5% water extracts of yeast‐fermented garlic (YF) or 1 µM Torin‐1 (T), in the presence or absence of 200 nM Bafilomycin A1 (Baf. A1) for 4 h. Whole cell lysate was used for western blot analysis with specific antibodies. Densitometry of the blots was performed using the FUSION SOLO S (Vilber Lourmat). (B) Validation of the *EGR1* deficient (KO) HAP1 cells. (C) Calculations for net LC3‐II flux levels in densitometric data of (A). The data was quantified as shown in Fig. 1D. (D) Confocal fluorescence microscopy. HAP1 *EGR1* WT and deficient (KO) cells expressing GFP‐LC3‐RFP were treated with 5% YF, 1 µM T, or 200 nM Baf.A1 for 4 h, followed by fixation using 2% paraformaldehyde. Fluorescence was visualized using confocal laser scanning microscopy (FV3000, Olympus). The bar indicates 10 µm. (D) Quantitation of autophagic flux. Cell treatments were the same as in (C). Cellular fluorescence intensity was measured using Cellometer Vision (Nexcelom Bioscience LLC) and FCS Express4 (De Novo software) was used for quantitative analysis. The data represent the mean ± SEM (*n* = 3). The letters indicate varying levels of significance analyzed with Tukey's test among the groups (*p < *0.05). SEM, standard error of the mean.

Since YF treatment altered *EGR1* expression, we investigated the role of *EGR1* in autophagy activation by examining *EGR1*‐deficient (knockout; KO) HAP1 cells. Results showed that *EGR1‐*KO cells treated with YF abolished the increase of LC3‐II in autophagy flux assay (Figure 3A–C, *p* < 0.05); however, Torin‐1 significantly increased LC3‐II accumulation upon *EGR1* depletion (Figure 3A, C, *p* < 0.05). Further, we confirmed the autophagy activity in HAP1 *EGR1‐*KO cells expressing GFP‐LC3‐RFP. Results showed that YF failed to significantly reduce the GFP/RFP ratio in HAP1 *EGR1‐*KO cells expressing GFP‐LC3‐RFP (Figure 3E *, p *> 0.05); however, Torin‐1 significantly reduced the GFP/RFP ratio. Moreover, GFP/RFP ratios significantly increased upon treatment with Bafilomycin A1 (Figure 3E *, p *< 0.05). The results suggest that the loss of *EGR1* did not affect mTOR‐mediated autophagy activation, but YF treatment required *EGR1* to increase autophagy activity.

### 3.4 Spermine (SPM)/Spermidine (SPD) Ratio Equivalent to YF Significantly Activated Autophagy

**FIGURE 4:**
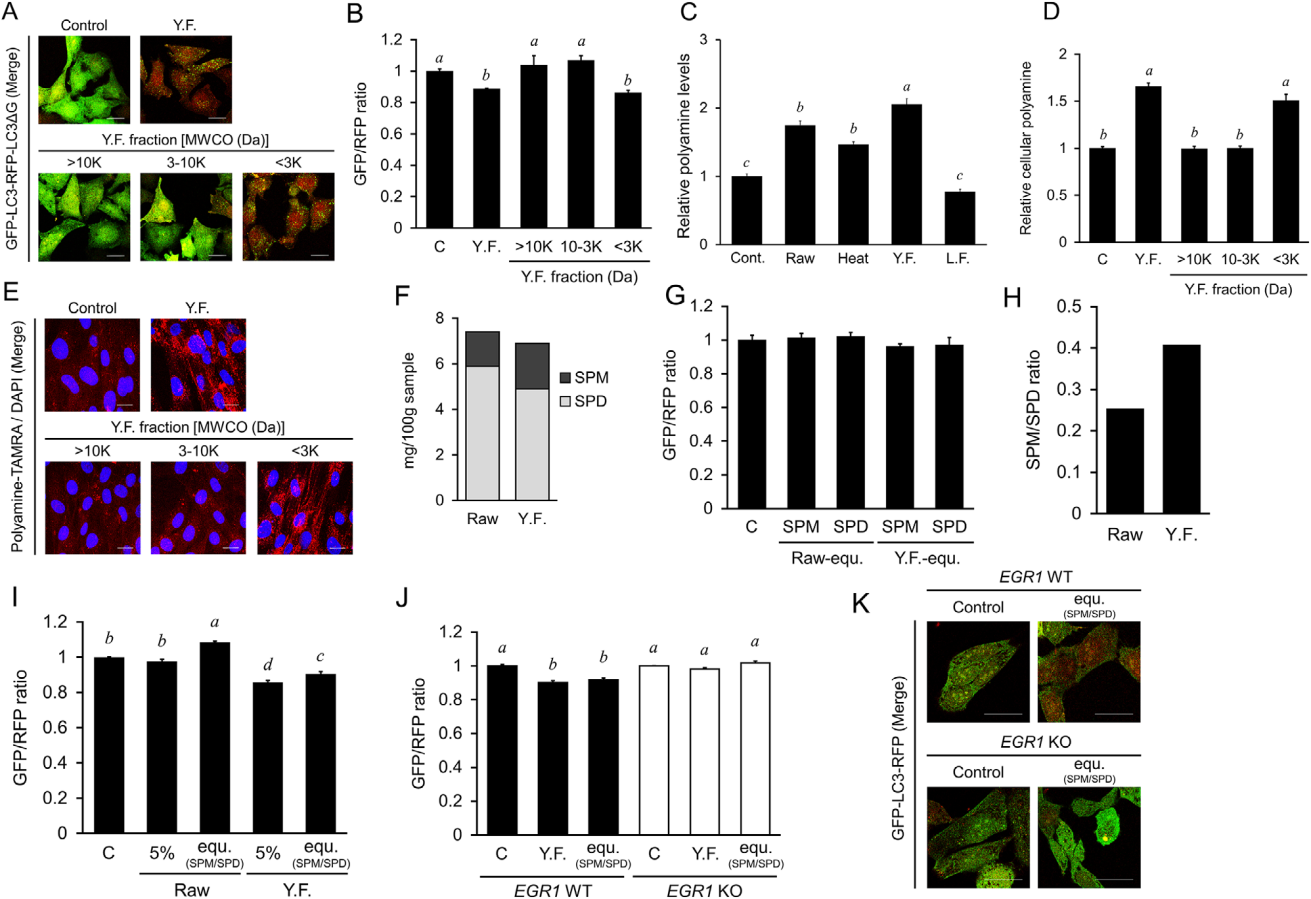
Bioactive fractions by ultrafiltration and quantification of polyamines in cells. (A, B) Determination of autophagic flux in yeast‐fermented garlic (YF) and ultrafiltration fractions of YF water extraction. (A) Confocal fluorescence microscopy. HeLa cells expressing GFP‐LC3‐RFP‐LC3ΔG were treated with 5% water extracts of YF, or molecular weight cutoff (MWCO) fractions by ultrafiltration, over 10 000 Da (>10k), 10 000–3000 Da (10k–3k), or under 3 000 Da (<3k) for 4 h, followed by fixation using 2% paraformaldehyde. (B) Quantitation of autophagic flux. Cell treatments were the same as in (A). (C, D) Quantification of cellular polyamines in extracts of different processed garlic. Relative cellular polyamine levels in extracts of different processed garlic. (C) Relative cellular polyamine levels of ultrafiltration fractions of YF water extracts. (D, E) PolyamineRED staining of polyamines in cells treated with YF and ultrafiltration fractions of YF water extracts. The treatment in OUMS‐36T‐1 cells was the same as in (A), followed by staining with 10 µM of PolyamineRED (Funakoshi) for 30 min. Fluorescence was visualized using confocal laser scanning microscopy (FV3000, Olympus), Scale bar indicates 20 µm. (F) The amount of spermine (SPM) and spermidine (SPD) in raw garlic (Raw) and YF. (G) Quantitation of autophagic flux. HeLa cells expressing GFP‐LC3‐RFP‐LC3ΔG were treated with 0.7 µM SPM or 4.0 µM SPD, equivalent to 5% Raw (Raw‐equ.), or 1.0 µM SPM or 3.4 µM SPD, equivalent to 5% YF (Y.F.‐equ.) for 4 h, assuming the entire amount of SPM and SPD were extracted from 5% Raw and YF. (H) The ratio of SPM/SPD in Raw and YF. (I) Quantitation of autophagic flux by SPM/SPD ratio. HeLa cells expressing GFP‐LC3‐RFP‐LC3ΔG were treated with 5% Raw, or 0.7 µM SPM and 4.0 µM SPD (equ. [SPM/SPD]), equivalent to 5% Raw, or 5% YF water extract, or 1.0 µM SPM and 3.4 µM SPD (equ. [SPM/SPD]), equivalent to 5% YF for 4 I. (J) Quantitation of autophagic flux related to *EGR1* gene after treatment with SPM/SPD ratio. HAP1 *EGR1* WT and KO cells expressing GFP‐LC3‐RFP were treated with 5% YF water extract, or 1.0 µM SPM and 3.4 µM SPD (equ. [SPM/SPD]), equivalent to 5% YF for 4 h. Cellular fluorescence intensity was measured using Cellometer Vision (Nexcelom Bioscience LLC) and FCS Express4 (De Novo software) was used for quantitative analysis (B, D, E). (K) Confocal fluorescence microscopy. Cell treatments were the same as in (E), followed by fixation using 2% paraformaldehyde. Fluorescence was visualized using confocal laser scanning microscopy (FV3000, Olympus). Bar indicates 20 µm. The data represent the mean ± SEM (*n* = 3). The letters indicate varying levels of significance (*p < *0.05) analyzed with Tukey's test among the groups after one‐way ANOVA. ANOVA, analysis of variance; SEM, standard error of the mean.

Since cells treated with YF presented enhanced autophagy flux independent to mTOR signaling, we further investigated the bioactive compounds in YF associated with autophagy induction. The components of YF were separated according to molecular weight using ultrafiltration, and allotted into 10 000 Da (>10k), 10 000–3000 Da (10k–3k), or under 3000 Da (<3k) groups, respectively. Cells were treated with 5% YF and with the three filtration‐derived groups. As shown in Figure 4A, B, the GFP/RFP ratio was significantly reduced in YF‐treated groups and the YF fractionation <3k group (*p *< 0.05), suggesting that the molecular weights of the bioactive compounds were under 3000 Da. Allicin is typically considered the bioactive compound in garlic that may affect autophagy activity. However, allicin is highly unstable and thermolabile, decomposing within hours after garlic is cut [21]. Our results further indicate that neither powdered nor raw garlic significantly increases autophagy activity. Therefore, we conclude that allicin, at the concentrations used or after garlic processing, does not affect autophagy activity due to its rapid decomposition. Additionally, fermentation food is known to significantly increase the polyamine content in foods [11, 22]. Therefore, we performed a polyamine quantitative assay, and results showed that YF had the highest polyamine content (Figure 4C, *p* < 0.05). Polyamine‐TAMRA/DAPI staining showed that polyamines were enriched in the YF fractionation <3k group (Figure 4D, E, *p *< 0.05), indicating that polyamines in YF may contribute to autophagy induction.

SPM and SPD are the principal polyamine components in YF (Figure 4F). SPM levels were higher in YF than that in Raw garlic, whereas SPD levels were lower. Cells were treated with amounts of SPM and SPD equivalent to those contained in the Raw or YF groups, to validate whether SPM or SPD contributed to autophagy activation observed during YF treatment. However, the GFP/RFP ratio did not differ significantly across all treatments (Figure 4G, *p *< 0.05). Subsequently, we treated the cells with an SPM/SPD ratio (1 µM:3.4 µM) equivalent to that in the Raw or YF groups (Figure 4H). The SPM/SPD ratio equivalent to Raw showed significantly higher GFP/RFP ratios (Figure 4I, *p* < 0.05), whereas the SPM/SPD ratio equivalent to YF displayed a significantly lower GFP/RFP ratio, suggesting that varying ratios of SPM/SPD modulated autophagy activity. The analysis utilized a regression method to optimize the SPM/SPD ratio in relation to the GFP/RFP ratio, as illustrated in Figures S3 and S4. The findings suggest that a derived SPM/SPD ratio of about 0.2 correlates with the lowest observed GFP/RFP ratio. This implies that a ratio of approximately 1:5 between SPM and SPD maximizes autophagy induction in HeLa cells.

Moreover, we investigated whether the ratio of SPM/SPD induced autophagy in an *EGR1‐*dependent manner. The results showed that *EGR1* WT cells treated with YF and the equivalent SPM/SPD ratio displayed significantly lower GFP/RFP ratios (Figure 4J, K, *p* < 0.05); however, *EGR1*‐deficient ((cells treated with YF and the equivalent SPM/SPD ratio displayed no significant difference in GFP/RFP ratio. These results indicate that the ratio of SPM/SPD induced autophagy in an *EGR1*‐dependent manner.

### 3.5 Spermine (SPM)/Spermidine (SPD) Ratio Equivalent to YF Increased EGR1 Expression In Vivo

**FIGURE 5:**
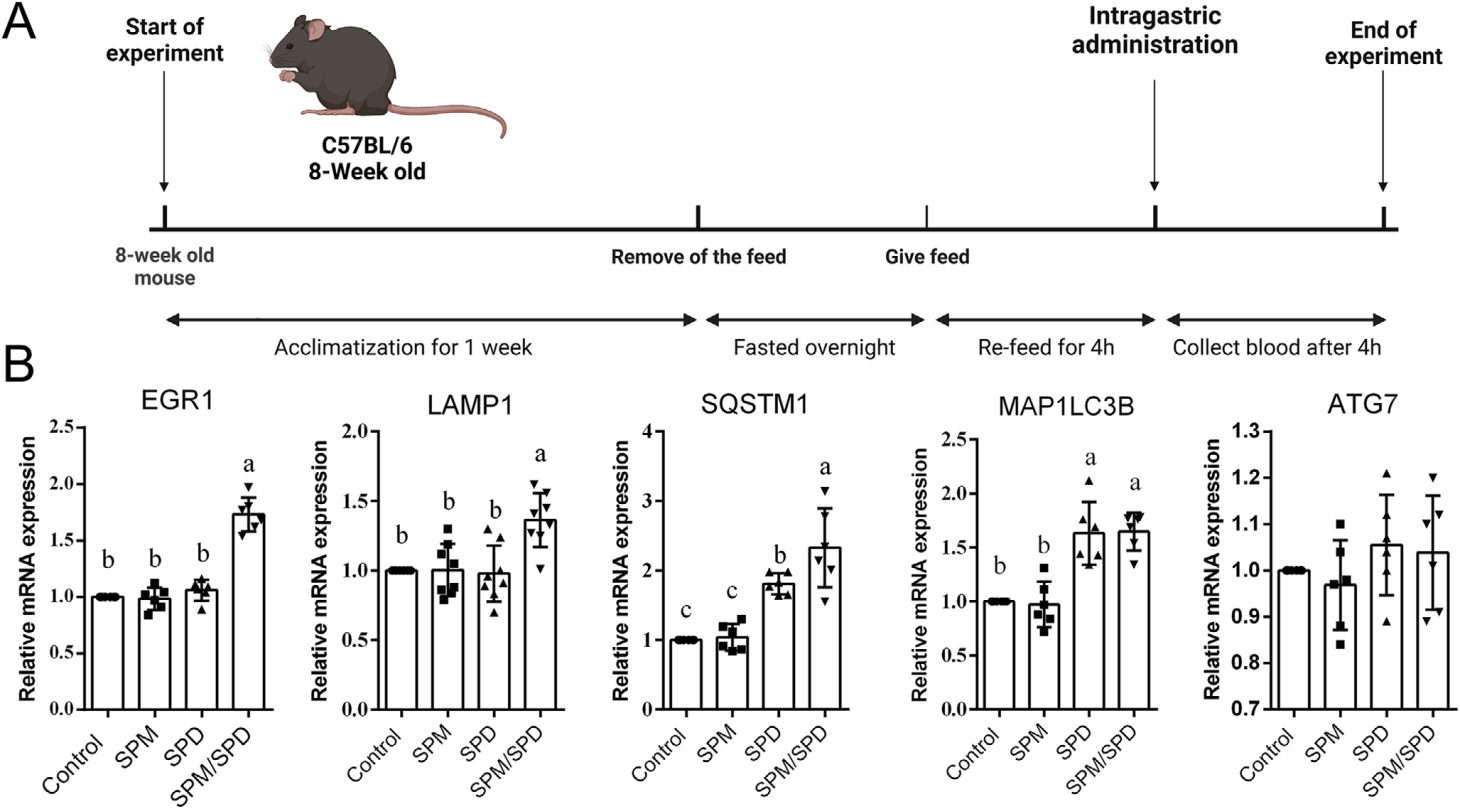
Spermine (SPM)/Spermidine (SPD) ratio equivalent to yeast‐fermented garlic (YF) increased EGR1 expression in vivo. (A) A scheme indicating the administration procedure of in vivo experiments. (B) Validation of *EGR1* targeted gene expression through qPCR. Mice blood RNA was isolated using a RiboPure‐Blood RNA Isolation Kit, following the manufacturer's instructions. Reverse transcription was performed using cDNA synthesis kits from Takara, following the manufacturer's manual. The samples were analyzed using real time PCR (ΔΔCt method). The data represent the mean ± SEM (*n* = 8). The letters indicate varying levels of significance (*p < *0.05) analyzed with Tukey's test among the groups after one‐way ANOVA. ANOVA, analysis of variance; SEM, standard error of the mean.

Although SPM/SPD balance was validated in vitro, it was crucial to determine if this balance would also affect EGR1 and autophagy‐related genes in living organisms. To confirm this in vivo, we performed intragastric administration of SPM, SPD, or a combined SPM/SPD at a ratio equivalent to YF in mice (Figure 5A). Mice were fasted and re‐fed before euthanized to avoid starvation or significant autophagy regulation. Following treatment, blood was collected from the eyeball, RNA was isolated from the blood samples, and reverse transcription was performed. The resulting cDNA was then used for qPCR analysis. The results demonstrated that the SPM/SPD combination significantly upregulated the relative expression of *EGR1* in mice compared to other treatments (Figure 5B, *p* < 0.05). Additionally, mice treated with the SPM/SPD combination exhibited significantly higher expression levels of *LAMP1*, *SQSTM1*, and *MAP1LC3B* (*p* < 0.05). In contrast, mice receiving SPD alone showed increased expression of *SQSTM1* and *MAP1LC3B* (*p* < 0.05), while the expression levels of *EGR1* and *LAMP1* remained unchanged compared to controls (*p* > 0.05).

### 3.6 Autophagy Activated by SPM/SPD Protected Against Proteostasis Stress In Vitro

**FIGURE 6:**
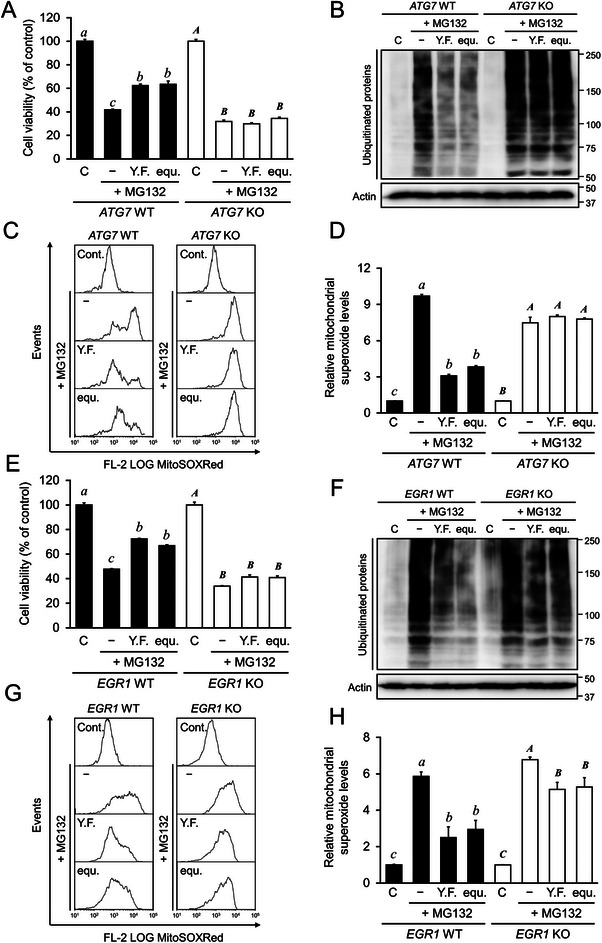
The effects of yeast‐fermented garlic (YF) and spermine/spermidine ratio equivalent to YF on cellular protection from MG132 proteostress in HeLa *ATG7* WT or deficient (KO) cells, or HAP1 *EGR1* WT or deficient (KO) cells. (A) Cell viability rate. HeLa *ATG7* WT or KO cells were pretreated with 5% YF water extracts or spermine/spermidine (1.0 µM/3.4 µM; equ.), equivalent to 5% YF for 12 h, followed by exposure to 10 µM MG132 for another 12 h. The cell viability rate was assessed using the MTT assay (Dojindo) and was expressed as the optical density ratio of the treatment to control. (B) Ubiquitinated proteins levels. Cell treatments were the same as in (A). Whole cell lysate was used for western blot analysis with specific antibodies. Detection of the blots was performed using the FUSION SOLO S (Vilber Lourmat). (C) Intensity histogram of mitochondrial superoxide production. Cell treatments were the same as in (A), followed by staining with 10 µM of MitoSOX RED (Invitrogen) for 1 h. Cellular fluorescence intensity was measured using Cellometer Vision (Nexcelom Bioscience LLC) and FCS Express4 (De Novo software) was used for quantitative analysis. (D) Calculations for relative mitochondrial superoxide levels in intensity data of (C). (E) The cell viability rate. HAP1 *EGR1* WT or deficient (KO) cells were pretreated with 5% YF water extracts or spermine/spermidine (1.0 µM/3.4 µM; equ.), equivalent to 5% YF for 12 h, followed by exposure to 10 µM MG132 for another 12 h. The cell viability rate was assessed using the MTT assay (Dojindo) and was expressed as the optical density ratio of the treatment to control. (F) Ubiquitinated protein levels. Cell treatments were the same as in (A). Whole‐cell lysates were used for western blot analysis with specific antibodies. Detection of the blots was performed using the FUSION SOLO S (Vilber Lourmat). (G) Intensity histogram of mitochondrial superoxide levels. Cell treatments were the same as in (A), followed by staining with 10 µM MitoSOX RED (Invitrogen) for 1 h. Cellular fluorescence intensity was measured with Cellometer Vision (Nexcelom Bioscience LLC) and FCS Express4 (De Novo software) was used for quantitative analysis. (H) Calculations for relative mitochondrial superoxide levels in intensity data of (G). The data represent the mean ± SEM (*n* = 3). The letters indicate varying levels of significance (*p < *0.05) analyzed with Tukey's test among the groups after one‐way ANOVA. ANOVA, analysis of variance; SEM, standard error of the mean.

Proteasome inhibition leads to proteins aggregation in the cytosol and induces cytotoxic unfolded proteins responses; elimination of unfolded protein is dependent on autophagy activation [23]. Thus, *ATG7* WT or *ATG7*‐KO cells were treated with MG132, a cell‐permeable proteasome inhibitor, to induce cytotoxic unfolded protein aggregation, and supplemented with or without YF or a YF‐equivalent SPM/SPD ratio to investigate the cytoprotective effects of autophagy. Results showed that *ATG7* WT cells treated with YF or YF‐equivalent SPM/SPD displayed significantly higher cell viability after MG132‐induced cytotoxic unfolded protein aggregation (Figure 6A, *p *< 0.05). However, *ATG7*‐KO cells displayed no significant difference in cell viability under the same conditions. Moreover, *ATG7* WT cells treated with MG132 and supplemented with YF or YF‐equivalent SPM/SPD displayed fewer ubiquitinated proteins (Figure 6B, *p* < 0.05), whereas ubiquitinated proteins showed no difference in *ATG7*‐KO cells among all treatments, indicating that YF or YF‐equivalent SPM/SPD‐activated autophagy reduced the MG132‐induced unfolded protein accumulation. Furthermore, *ATG7* WT cells treated with YF or YF‐equivalent SPM/SPD showed lower mitochondrial superoxide red signaling in living cells (Figure 6C, *p* < 0.05), whereas no difference was detected in *ATG7*‐KO cells (Figure S5A). Moreover, relative mitochondrial superoxide levels appeared significantly reduced in *ATG7* WT cells treated with YF or YF‐equivalent SPM/SPD (Figure 6D, *p* < 0.05), but no significant difference was observed in *ATG7*‐KO cells (*p* > 0.05). These data suggest that YF or YF‐equivalent SPM/SPD supplementation activated autophagy to reduce unfolded protein accumulation and mitochondrial superoxide generation.

Similarly, we used *EGR1* WT or *EGR1*‐deficient (KO) cells treated with MG132 to induce cytotoxic unfolded protein aggregation and supplemented with or without YF or YF‐equivalent SPM/SPD to confirm whether the induced autophagy was dependent on *EGR1* activation. *EGR1* WT cells treated with YF or YF‐equivalent SPM/SPD displayed relatively higher cell viability in response to MG132‐induced cytotoxicity (Figure 6E, *p* < 0.05), whereas *EGR1*‐deficient (KO) cells showed no significant difference under the same conditions. Furthermore, *EGR1* WT cells treated with MG132 and supplemented with YF or YF‐equivalent SPM/SPD displayed fewer ubiquitinated proteins (Figure 6F, *p* < 0.05), whereas *EGR1*‐deficient (KO) cells exhibited no significant differences under the same conditions. These results indicate that YF or YF‐equivalent SPM/SPD activated autophagy in an *EGR1*‐dependent manner. Moreover, *EGR1* WT cells treated with YF or YF‐equivalent SPM/SPD also exhibited lower mitochondrial superoxide levels (Figure 6G, H, *p* < 0.05), whereas *EGR1*‐deficient (KO) cells showed no significant difference (Figure S5B).

## 4 Discussion

### 4.1 YF Supplementation Induced mTOR Independent Autophagy

Autophagy is an important intracellular degradation system generally activated via inhibition of mTOR signaling in response to nutrition starvation. mTOR is a master regulator of cellular metabolism, including cell growth, proliferation, and protein synthesis [24], its critical role under physiological conditions, which makes mTOR a challenging therapeutic target [6]. Research has shown that autophagy activation without impaired mTOR signaling may promote component renewal while maintaining normal cellular function [25]. This study showed that dietary supplementation with YF‐derived SPM and SPD activated autophagy without disrupting phospho‐p70‐S6K and 4EBP1, indicating that autophagy activation occurred independently of mTOR signaling.

### 4.2 SPD and SPM Supplementation Induced EGR1‐Mediated Autophagy

Polyamine concentrations in mammals are determined by nutritional supply and internal metabolism, including synthesis by the intestinal microbiota, uptake from diet, cellular biosynthesis, amino acid metabolism, and urinary excretion. Inhibition of polyamine biosynthesis has been shown to arrest the cell cycle, while provision of exogenous polyamines restores cell growth [26]. Tissue SPD concentrations decline with age in model organisms and humans [27, 28, 29]. This is due to a decline in the biosynthetic activities of polyamine‐producing enzymes [30]. Dietary SPD supplementation ameliorates age‐induced memory impairment in flies and protects against autoimmune‐directed demyelination of neurons in a mouse model of multiple sclerosis [31, 32]. SPM also reduced the growth of transplantable tumors, stimulated anti‐cancer immune surveillance in combination with chemotherapy, and suppressed tumorigenesis induced by chemical insult in mice [33]. Increased levels of intracellular SPD and SPM are associated with lower mortality rates from cardiovascular diseases and cancer, according to human epidemiological studies [33]. At the molecular levels, SPD is known to act as a substrate for the hypusination reaction, which activates eIF5A and thereby promotes the translation and synthesis of the pro‐autophagic transcription factor *TFEB* and mitochondrial biogenesis [34]. Concurrently, SPM has been shown to increase autolysosomal flux through lysosome enhancement, thereby aiding in the clearance of aggregates [35]. A study involving humans has demonstrated that the combined intake of SPM and SPD can modulate T‐cell function in older adults through autophagy, though the precise mechanisms are still unclear [36]. Our research contributes a novel insight, appears that *EGR1* may play a pivotal role in this enhanced autophagy induction by the synergistic effect of SPM and SPD. Recent studies have indicated that *EGR1* is a positive transcriptional regulator of *TFEB* expression. With deficient of *EGR1*, the *TFEB*‐mediated transcriptional response to starvation is compromised [37], suggesting that *EGR1* is a critical transcriptional factor for initiating *TFEB*‐mediated autophagy.

### 4.3 The SPM/SPD Ratio Significantly Shift Autophagy Flux In Vitro

Our study found that a specific ratio (1 µM:3.4 µM) of SPM/SPD supplementation significantly increased autophagy induction compared to individual SPD or SPM. Furthermore, promising results were observed in a proof‐of‐concept trial, which reported that 3 months of supplementation with a plant extract predominantly containing SPD and SPM (providing 1.2 mg of SPD and 0.6 mg of SPM per 1 g of extract) was not only safe but also improved memory functions in older adults with subjective cognitive decline [38, 39]. Notably, a metabolic profile indicated that the SPM and SPD ratio declines with age [22]. SPD supplementation restored this pathway and improved the responses of B cells in the elderly [40]. Depletion of cellular SPD and SPM by overexpression of a polyamine catabolic enzyme caused a rapid arrest in protein synthesis and cell growth [41]. Further investigations revealed that in aged mice, the total and free intracellular concentrations of SPD in CD8+ T cells were roughly half of those found in younger mice [42]. Aged CD8+ T cells also exhibited impaired mitochondrial and cytotoxic functions, supporting the hypothesis that immune cell functionality correlates positively with cellular polyamine concentrations. In vivo supplementation appeared to enhance *EGR1* target gene expression in mouse blood, including *MAP1LC3B* and *SQSTM1*, indicating increased autophagy activity in white blood cells and potentially improving immune functions. The mitogen‐activated protein kinase pathway, along with other intracellular signaling cascades, has been implicated in the transcriptional regulation of *EGR1* [43]. Additionally, polyamines are known to modulate various signaling pathways that govern cell proliferation and differentiation [44], and they have been specifically linked to the activation of the PI3K/Akt pathway [44, 45]. Our findings align with this, showing increased phosphorylation of S6K and 4EBP1, indicative of enhanced proliferative signaling. Moreover, polyamine metabolism generates hydrogen peroxide, which plays a crucial role in reactive oxygen species (ROS) signaling. Since ROS has been shown to induce *EGR1* expression through stress‐activated protein kinase/c‐Jun N‐terminal kinase and mitogen‐activated protein kinase/extracellular signal‐regulated kinase pathways, it is likely that ROS contributes to the upregulation of *EGR1* observed in response to polyamines. However, further investigation is necessary to confirm the involvement of these signaling pathways in polyamine‐induced *EGR1* expression. Furthermore, *EGR1* as an important transcription factor that controls memory formation and brain neuron reprogramming showed an age‐related reduction [46]. Moreover, *EGR1* expression is shifted forward with age in male mice, *EGR1* deletion accelerates liver age‐related metabolic dysfunction [47]. *EGR1* has been suggested to act upstream of other aging‐related genes and appears to be pro‐apoptotic, However, its precise role in human aging is still under investigation. The relationship between internal polyamine levels and *EGR1* expression suggests a link to aging‐related alterations. In our studies, treating OUMS‐36T‐1 cells with YF significantly boosted *EGR1* expression, and YF‐induced autophagy was found to depend on *EGR1* signaling. However, while *EGR1* knockdown dramatically reduced the transcription of numerous autophagy‐related genes, *EGR1* overexpression only modestly affected these genes [48].

### 4.4 SPM/SPD‐Activated Autophagy Protected Cellular Proteostasis

Global impairment of proteostasis is a vital outcome of compromised protein degradation in cells and constitutes a major hallmark of aging [49]. Clearance of damaged DNA, precise calorie restriction, and maintenance of mitochondrial health are the key functions accomplished by the ubiquitin‐proteasome system (UPS) and autophagy to slow down the overall progress of age‐associated changes [50]. To decrease the burden on the UPS, autophagy promotes the selective degradation of proteins in a tightly regulated manner to improve the physiological balance of cellular proteostasis that may become disrupted due to the accumulation of misfolded proteins [51]. Previous studies reported that SPM increased the acetylation of microtubules to facilitate retrograde transport of autophagosomes from the cellular periphery to lysosomes, and thus facilitated autophagic degradation of misfolded protein aggregates [35]. Dietary SPD supplementation induced autophagy in the mouse brain and maintained mitochondrial stability [52]. Consistently, our study demonstrated that YF‐derived SPM and SPD supplementation reduced ubiquitinated protein aggregates via activation of *EGR1*‐dependent autophagy, decreased mitochondrial oxidative stress, and ameliorated the accumulation of harmful proteins.

## 5 Conclusion

Collectively, our study revealed that YF‐derived SPM and SPD supplementation activated mTORC1‐independent, *EGR1*‐dependent autophagy. YF promoted autophagy, consequently reduced ubiquitinated proteins, and maintained mitochondrial homeostasis. Moreover, in vivo evidence showed that supplementation of SPM and SPD with a ratio equivalent to YF increased *EGR1*‐targeted gene expression in mouse blood RNA, suggesting that supplementation with SPM and SPD may restore aging‐induced autophagy and alleviate immune suppression. Our findings demonstrate that dietary SPM and SPD supplementation may ameliorate aging‐associated disease and protein aggregation by promoting autophagy.

## Conflicts of Interest

The authors declare no conflicts of interest.

## Supporting information



Supporting Information

## Data Availability

The data that support the findings of this study are available from the corresponding author upon reasonable request.

## References

[1] M. P. Mattson , V. D. Longo , and M. Harvie , “Impact of Intermittent Fasting on Health and Disease Processes,” Ageing Research Reviews 39 (2017): 46–58. 27810402 10.1016/j.arr.2016.10.005 PMC5411330

[2] C. Franceschi , P. Garagnani , P. Parini , C. Giuliani , and A. Santoro , “Inflammaging: A New Immune‐Metabolic Viewpoint for Age‐Related Diseases,” Nature Reviews Endocrinology 14 (2018): 576–590. 10.1038/s41574-018-0059-4 30046148

[3] G. Kroemer and B. Levine , “Autophagic Cell Death: The Story of a Misnomer,” Nature Reviews Molecular Cell Biology 9 (2008): 1004–1010. 18971948 10.1038/nrm2527 PMC2727358

[4] D. C. Rubinsztein , G. Marino , and G. Kroemer , “Autophagy and Aging,” Cell 146 (2011): 682–695. 21884931 10.1016/j.cell.2011.07.030

[5] Y. Aman , T. Schmauck‐Medina , M. Hansen , et al., “Autophagy in Healthy Aging and Disease,” Nature Aging 1 (2021): 634–650. 34901876 10.1038/s43587-021-00098-4 PMC8659158

[6] Y. C. Kim and K. L. Guan , “mTOR: A Pharmacologic Target for Autophagy Regulation,” Journal of Clinical Investigation 125 (2015): 25–32. 25654547 10.1172/JCI73939 PMC4382265

[7] Y. Kabeya , N. Mizushima , T. Ueno , et al., “LC3, a Mammalian Homologue of Yeast Apg8p, Is Localized in Autophagosome Membranes After Processing,” The EMBO Journal 19 (2000): 5720–5728. 11060023 10.1093/emboj/19.21.5720 PMC305793

[8] T. Kaizuka , H. Morishita , Y. Hama , et al., “An Autophagic Flux Probe That Releases an Internal Control,” Molecular Cell 64 (2016): 835–849. 27818143 10.1016/j.molcel.2016.09.037

[9] S. V. Rana , R. Pal , K. Vaiphei , S. K. Sharma , and R. P. Ola , “Garlic in Health and Disease,” Nutrition Research Reviews 24 (2011): 60–71. 24725925 10.1017/S0954422410000338

[10] C. Zhang , X. He , Y. Sheng , et al., “Allicin Regulates Energy Homeostasis Through Brown Adipose Tissue,” iScience 23 (2020): 101113. 32413611 10.1016/j.isci.2020.101113 PMC7226876

[11] Y. Feng , C. Ping Tan , C. Zhou , et al., “Effect of Freeze‐Thaw Cycles Pretreatment on the Vacuum Freeze‐Drying Process and Physicochemical Properties of the Dried Garlic Slices,” Food Chemistry 324 (2020): 126883. 32344350 10.1016/j.foodchem.2020.126883

[12] B. N. do Carmo Brito , R. Campos Chiste , R. da Silva Pena , M. B. Abreu Gloria , and A. Santos Lopes , “Bioactive Amines and Phenolic Compounds in Cocoa Beans Are Affected by Fermentation,” Food Chemistry 228 (2017): 484–490. 28317753 10.1016/j.foodchem.2017.02.004

[13] L. Bayan , P. H. Koulivand , and A. Gorji , “Garlic: A Review of Potential Therapeutic Effects,” Avicenna Journal of Phytomedicine 4 (2014): 1–14. 25050296 PMC4103721

[14] C. Moinard , L. Cynober , and J. P. de Bandt , “Polyamines: Metabolism and Implications in Human Diseases,” Clinical Nutrition 24 (2005): 184–197. 15784477 10.1016/j.clnu.2004.11.001

[15] A. E. Pegg , “Mammalian Polyamine Metabolism and Function,” IUBMB Life 61 (2009): 880–894. 19603518 10.1002/iub.230 PMC2753421

[16] T. Eisenberg , M. Abdellatif , S. Schroeder , et al., “Cardioprotection and Lifespan Extension by the Natural Polyamine Spermidine,” Nature Medicine 22 (2016): 1428–1438. 10.1038/nm.4222 PMC5806691 27841876

[17] F. Madeo , T. Eisenberg , F. Pietrocola , and G. Kroemer , “Spermidine in Health and Disease,” Science 359 (2018): eaan2788. 29371440 10.1126/science.aan2788

[18] F. Madeo , M. A. Bauer , D. Carmona‐Gutierrez , and G. Kroemer , “Spermidine: A Physiological Autophagy Inducer Acting as an Anti‐Aging Vitamin in Humans?” Autophagy 15 (2019): 165–168. 30306826 10.1080/15548627.2018.1530929 PMC6287690

[19] C. Y. Liao , O. M. P. Kummert , A. M. Bair , et al., “The Autophagy Inducer Spermidine Protects Against Metabolic Dysfunction During Overnutrition,” Journals of Gerontology Series A, Biological Sciences and Medical Sciences 76 (2021): 1714–1725. 34060628 10.1093/gerona/glab145 PMC8436989

[20] C. H. Jung , S. H. Ro , J. Cao , N. M. Otto , and D. H. Kim , “Mtor Regulation of Autophagy,” FEBS Letters 584 (2010): 1287–1295. 20083114 10.1016/j.febslet.2010.01.017 PMC2846630

[21] L. D. Lawson and C. D. Gardner , “Composition, Stability, and Bioavailability of Garlic Products Used in a Clinical Trial,” Journal of Agricultural and Food Chemistry 53 (2005): 6254–6261. 16076102 10.1021/jf050536+ PMC2584604

[22] S. Saiki , Y. Sasazawa , M. Fujimaki , et al., “A Metabolic Profile of Polyamines in Parkinson Disease: A Promising Biomarker,” Annals of Neurology 86 (2019): 251–263. 31155745 10.1002/ana.25516 PMC6772170

[23] M. Fakruddin , F. Y. Wei , T. Suzuki , et al., “Defective Mitochondrial tRNA Taurine Modification Activates Global Proteostress and Leads to Mitochondrial Disease,” Cell Reports 22 (2018): 482–496. 29320742 10.1016/j.celrep.2017.12.051

[24] R. A. Saxton and D. M. Sabatini , “mTOR Signaling in Growth, Metabolism, and Disease,” Cell 169 (2017): 361–371. 10.1016/j.cell.2017.03.035 28388417

[25] M. J. Munson and I. G. Ganley , “MTOR, PIK3C3, and Autophagy: Signaling the Beginning From the End,” Autophagy 11 (2015): 2375–2376. 26565689 10.1080/15548627.2015.1106668 PMC4835211

[26] O. Heby , “Role of Polyamines in the Control of Cell Proliferation and Differentiation,” Differentiation 19 (1981): 1–20. 6173280 10.1111/j.1432-0436.1981.tb01123.x

[27] T. Eisenberg , H. Knauer , A. Schauer , et al., “Induction of Autophagy by Spermidine Promotes Longevity,” Nature Cell Biology 11 (2009): 1305–1314. 19801973 10.1038/ncb1975

[28] V. K. Gupta , L. Scheunemann , T. Eisenberg , et al., “Restoring Polyamines Protects From Age‐Induced Memory Impairment in an Autophagy‐Dependent Manner,” Nature Neuroscience 16 (2013): 1453–1460. 23995066 10.1038/nn.3512

[29] G. Scalabrino and M. E. Ferioli , “Polyamines in Mammalian Ageing: An Oncological Problem, Too? A Review,” Mechanisms of Ageing and Development 26 (1984): 149–164. 6384679 10.1016/0047-6374(84)90090-3

[30] K. Nishimura , R. Shiina , K. Kashiwagi , and K. Igarashi , “Decrease in Polyamines With Aging and Their Ingestion From Food and Drink,” Journal of Biochemistry 139 (2006): 81–90. 16428322 10.1093/jb/mvj003

[31] K. Abe , N. Chida , N. Nishiyama , and H. Saito , “Spermine Promotes the Survival of Primary Cultured Brain Neurons,” Brain Research 605 (1993): 322–326. 8481782 10.1016/0006-8993(93)91759-l

[32] G. Laube , H. G. Bernstein , G. Wolf , and R. W. Veh , “Differential Distribution of Spermidine/Spermine‐Like Immunoreactivity in Neurons of the Adult Rat Brain,” Journal of Comparative Neurology 444 (2002): 369–386. 11891649 10.1002/cne.10157

[33] F. Pietrocola , F. Castoldi , O. Kepp , et al., “Spermidine Reduces Cancer‐Related Mortality in Humans,” Autophagy 15 (2019): 362–365. 30354939 10.1080/15548627.2018.1539592 PMC6333461

[34] S. J. Hofer , A. K. Simon , M. Bergmann , et al., “Mechanisms of Spermidine‐Induced Autophagy and Geroprotection,” Nature Aging 2 (2022): 1112–1129. 37118547 10.1038/s43587-022-00322-9

[35] K. Phadwal , D. Kurian , M. K. F. Salamat , et al., “Spermine Increases Acetylation of Tubulins and Facilitates Autophagic Degradation of Prion Aggregates,” Scientific Reports 8 (2018): 10004. 29968775 10.1038/s41598-018-28296-y PMC6030104

[36] M. Fischer , J. Ruhnau , J. Schulze , et al., “Spermine and Spermidine Modulate T‐Cell Function in Older Adults With and Without Cognitive Decline Ex Vivo,” Aging (Albany NY) 12 (2020): 13716–13739. 32603310 10.18632/aging.103527 PMC7377836

[37] M. Cesana , G. Tufano , F. Panariello , et al., “EGR1 Drives Cell Proliferation by Directly Stimulating TFEB Transcription in Response to Starvation,” PLoS Biology 21 (2023): e3002034. 36888606 10.1371/journal.pbio.3002034 PMC9994711

[38] C. Schwarz , S. Stekovic , M. Wirth , et al., “Safety and Tolerability of Spermidine Supplementation in Mice and Older Adults With Subjective Cognitive Decline,” Aging (Albany NY) 10 (2018): 19–33. 29315079 10.18632/aging.101354 PMC5807086

[39] M. Wirth , G. Benson , C. Schwarz , et al., “The Effect of Spermidine on Memory Performance in Older Adults at Risk for Dementia: A Randomized Controlled Trial,” Cortex 109 (2018): 181–188. 30388439 10.1016/j.cortex.2018.09.014

[40] H. Zhang , G. Alsaleh , J. Feltham , et al., “Polyamines Control Eif5a Hypusination, TFEB Translation, and Autophagy to Reverse B Cell Senescence,” Molecular Cell 76 (2019): 110–125.e9. 31474573 10.1016/j.molcel.2019.08.005 PMC6863385

[41] S. Mandal , A. Mandal , H. E. Johansson , A. V. Orjalo , and M. H. Park , “Depletion of Cellular Polyamines, Spermidine and Spermine, Causes a Total Arrest in Translation and Growth in Mammalian Cells,” Proceedings of the National Academy of Sciences of the United States of America 110 (2013): 2169–2174. 23345430 10.1073/pnas.1219002110 PMC3568356

[42] M. Al‐Habsi , K. Chamoto , K. Matsumoto , et al., “Spermidine Activates Mitochondrial Trifunctional Protein and Improves Antitumor Immunity in Mice,” Science 378 (2022): eabj3510. 36302005 10.1126/science.abj3510

[43] J. Shan , E. Dudenhausen , and M. S. Kilberg , “Induction of Early Growth Response Gene 1 (EGR1) by Endoplasmic Reticulum Stress Is Mediated by the Extracellular Regulated Kinase (ERK) Arm of the MAPK Pathways,” Biochimica et Biophysica Acta (BBA)—Molecular Cell Research 1866 (2019): 371–381. 30290239 10.1016/j.bbamcr.2018.09.009 PMC6311436

[44] U. Bachrach , Y. C. Wang , and A. Tabib , “Polyamines: New Cues in Cellular Signal Transduction,” News in Physiological Sciences 16 (2001): 106–109. 11443226 10.1152/physiologyonline.2001.16.3.106

[45] J. G. C. Peeters , L. W. Picavet , S. G. J. M. Coenen , et al., “Transcriptional and Epigenetic Profiling of Nutrient‐Deprived Cells to Identify Novel Regulators of Autophagy,” Autophagy 15 (2019): 98–112. 30153076 10.1080/15548627.2018.1509608 PMC6287694

[46] Z. Sun , X. Xu , J. He , et al., “EGR1 Recruits TET1 to Shape the Brain Methylome During Development and Upon Neuronal Activity,” Nature Communications 10 (2019): 3892. 10.1038/s41467-019-11905-3 PMC6715719 31467272

[47] J. Wu , D. Bu , H. Wang , et al., “The Rhythmic Coupling of Egr‐1 and Cidea Regulates Age‐Related Metabolic Dysfunction in the Liver of Male Mice,” Nature Communications 14 (2023): 1634. 10.1038/s41467-023-36775-8 PMC10038990 36964140

[48] J. G. C. Peeters , L. W. Picavet , S. Coenen , et al., “Transcriptional and Epigenetic Profiling of Nutrient‐Deprived Cells to Identify Novel Regulators of Autophagy,” Autophagy 15 (2019): 98–112. 30153076 10.1080/15548627.2018.1509608 PMC6287694

[49] M. Martinez‐Vicente , G. Sovak , and A. M. Cuervo , “Protein Degradation and Aging,” Experimental Gerontology 2005, 40, 622–633. 16125351 10.1016/j.exger.2005.07.005

[50] L. Garcia‐Prat , M. Martinez‐Vicente , E. Perdiguero , et al., “Autophagy Maintains Stemness by Preventing Senescence,” Nature 2016, 529, 37–42. 26738589 10.1038/nature16187

[51] V. Joshi , A. Upadhyay , V. K. Prajapati , and A. Mishra , “How Autophagy Can Restore Proteostasis Defects in Multiple Diseases?” Medicinal Research Reviews 2020, 40, 1385–1439. 32043639 10.1002/med.21662

[52] M. Maglione , G. Kochlamazashvili , T. Eisenberg , et al., “Spermidine Protects From Age‐Related Synaptic Alterations at Hippocampal Mossy Fiber‐CA3 Synapses,” Scientific Reports 2019, 9, 19616. 31873156 10.1038/s41598-019-56133-3 PMC6927957

