# Supplementary material for: The Yeast‐Fermented Garlic and a Balance of Spermine/Spermidine Activates Autophagy via EGR1 Transcriptional Factor
Source: Mol Nutr Food Res. 2025 Feb 13;69(5):e202400606. doi: 10.1002/mnfr.202400606 (PMC11874185; doi:10.1002/mnfr.202400606)
Supplement: Supplementary file 1 — Supporting Information [file MNFR-69-e202400606-s001.docx]

**Garlic Processing Methods**

**Raw Garlic**

Fresh garlic cloves were used as the starting material. The cloves were peeled and ground into a fine paste using a commercial grinder. This paste was then freeze-dried to remove moisture. The resulting product was ground into a fine powder, yielding a 35% yield rate.

**Heated Garlic**

Fresh garlic cloves were processed similarly to the raw garlic method. After the garlic was ground into a paste, it underwent a sterilization process by heating at 85°C for 30 minutes. The sterilized paste was then freeze-dried and subsequently ground into a fine powder, also achieving a 35% yield rate.

**Lactic Acid Bacteria Fermentation**

Fresh garlic cloves were ground into a paste and sterilized by heating at 85°C for 30 minutes. Following sterilization, lactic acid bacteria and glucose were added to the paste. The mixture was then incubated at 35°C for 3 days, targeting a pH decrease from 6.2 to 4.6. After fermentation, the mixture was freeze-dried and ground into a fine powder, achieving a 36% yield rate.

**Unheated Lactic Acid Bacteria Fermentation**

Fresh garlic cloves were ground into a paste, to which lactic acid bacteria and glucose were added without prior sterilization. The mixture was incubated at 35°C for 3 days, aiming for a pH decrease from 6.2 to 5.5. Observations revealed that the strong garlic odor persisted and the pH did not decrease as expected, remaining above 5.4 even after a week of fermentation. Despite the lack of fermentation progress, the mixture was freeze-dried and ground into a fine powder, yielding a 36% yield rate.

**Yeast Fermentation Garlic**

Fresh garlic cloves were processed into a paste and sterilized by heating at 85°C for 30 minutes. Yeast and glucose were added to the sterilized paste, and the mixture was incubated at 35°C for 3 days, targeting a pH decrease from 6.2 to 6.3. After fermentation, the mixture was heated again at 85°C for 30 minutes, freeze-dried, and ground into a fine powder, achieving a 36% yield rate.

**Unheated Yeast Fermentation Garlic**

Fresh garlic cloves were ground into a paste, to which yeast and glucose were added without prior sterilization. The mixture was incubated at 35°C for 3 days, aiming for a pH decrease from 6.2 to 6.3. After fermentation, the mixture was ground into a fine powder, achieving a 35% yield rate.





**Supplementary Figure 1. Cell viability by using MTT or CCK8 assay and was expressed as the optical density ratio of the treatment to control.**

**(A)** Hela cells were treated with 5–10% water extracts of raw garlic (Raw), heated garlic (Heat), yeast-fermented garlic (YF), or *Lactobacillus*-fermented garlic (LF) for 24 h. **(B)** HAP1 cells were treated with 5–10% water extracts of raw garlic (Raw), heated garlic (Heat), yeast-fermented garlic (YF), or *Lactobacillus*-fermented garlic (LF) for 24 h. **(C)** Cell viability of OUMS-36T-1 cells treated as indicated above and determined by CCK8 assay. **(D)** Cell viability of Hela cells treated as indicated above and determined by CCK8 assay. **(E)** Cell viability of HAP1 cells treated as indicated above and determined by CCK8 assay.

The results revealed that all experiments using the CCK-8 assay displayed a significant increase in cell proliferation following the specified treatments. Conversely, the MTT assay detected no significant differences among the treatments. This discrepancy can be attributed to the fact that the CCK-8 assay measures the activity of most cellular dehydrogenases, while the MTT assay is specific to mitochondrial dehydrogenases. Polyamines, which influence cell signaling via phosphorylation and enhance cell metabolism, may selectively affect these enzymes, thus explaining the higher proliferation rates observed in the CCK-8 assay.


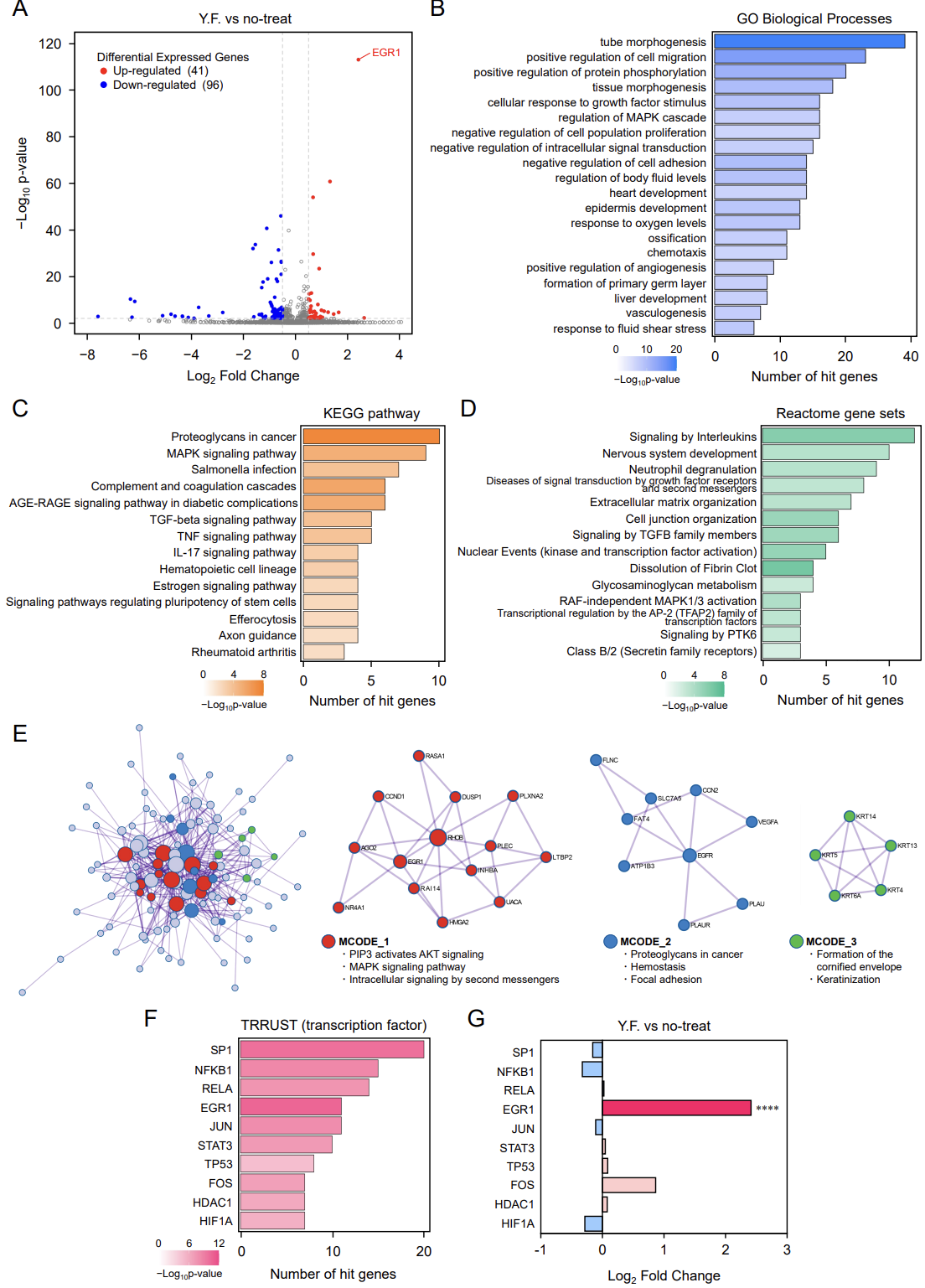


**Supplementary Figure 2. Transcriptional profile of OUMS-36T-1 cells treated with 5% water extracts of yeast-fermented garlic (YF) for 4 h.**

**(A)** Volcano plot **(B)** GO biological processes assay **(C)** KEGG pathway analysis **(D)** Reactome gene sets. **(E)** protein-protein interation analysis. **(F)** TRRUST transcriptional factor prediction assay. **(G)** DEGs of Top 10 Log2 Fold changes.


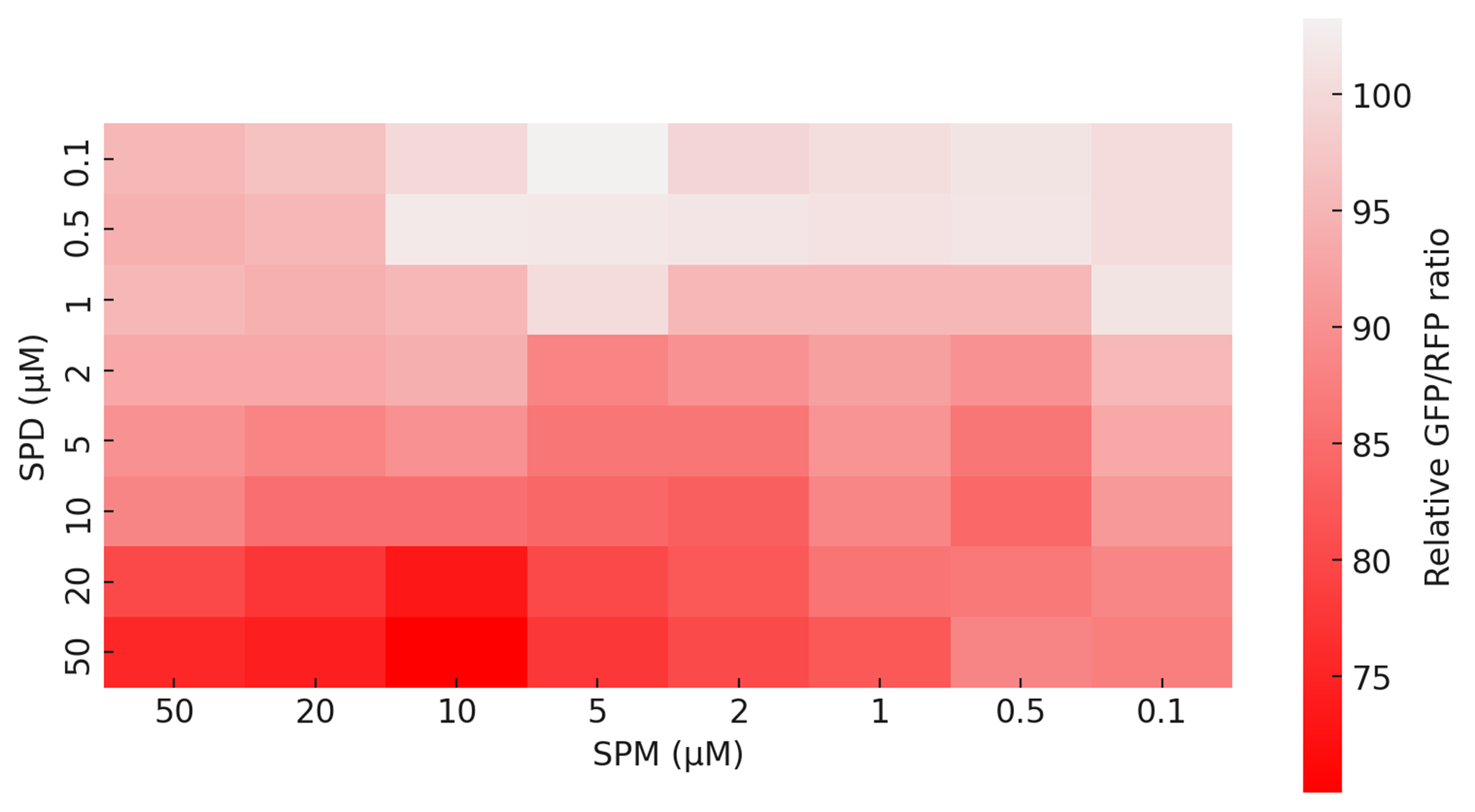


**Supplementary Figure 3.** Heatmap Analysis of SPD/SPM ratios Relative to GFP/RFP Ratios in Hela-GFP-LC3-RFP Cells.

**Regression equation:**

GFP/RFP ratio=2223.434×(SPM/SPD ratio)^2^ − 909.785×(SPM/SPD ratio)+ 154.36

Quadratic term coefficient (a): 2223.434

Linear term coefficient (b): −909.785

Intercept (c): 154.36

R square: 0.443

**Supplementary Figure 4. Prediction curve of SPM/SPD ratio to Relative GFP/RFP ratio in Hela-GFP-LC3-RFP cells. (A)** Figure of regression equation, The red point indicates the minimum value of 61.29472929 at the SPM/SPD ratio of approximately 0.20459, **(B)** Cutoff regression equation, the green dashed line represents the maximum cap of 100 for the GFP/RFP ratio.


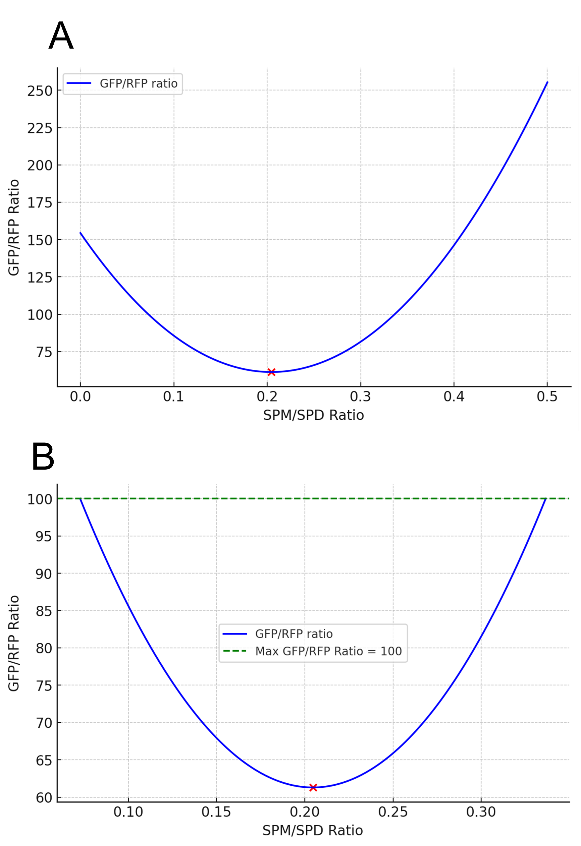


The regression models were constructed and evaluated using Python's scikit-learn library. The LinearRegression and PolynomialFeatures functions were used to implement linear and polynomial regression, respectively. The fitness for each model was assessed using the R-squared statistic, which measures the proportion of variance in the dependent variable that is predictable from the independent variable. The best-fitting model was selected based on the lowest mean squared error (MSE) calculated during model evaluation. The optimal concentration ratio, indicated by the minimum point on the fitted curve of the model (quadratic), was calculated to recommend the most effective combination of SPD and SPM for enhancing autophagy activity. Regression results were visualized by plotting the predicted GFP/RFP ratios against the SPM/SPD ratios using matplotlib in Python. This plot provides a visual representation of the model's accuracy in predicting relative GFP/RFP ratio across different SPM/SPD ratios.


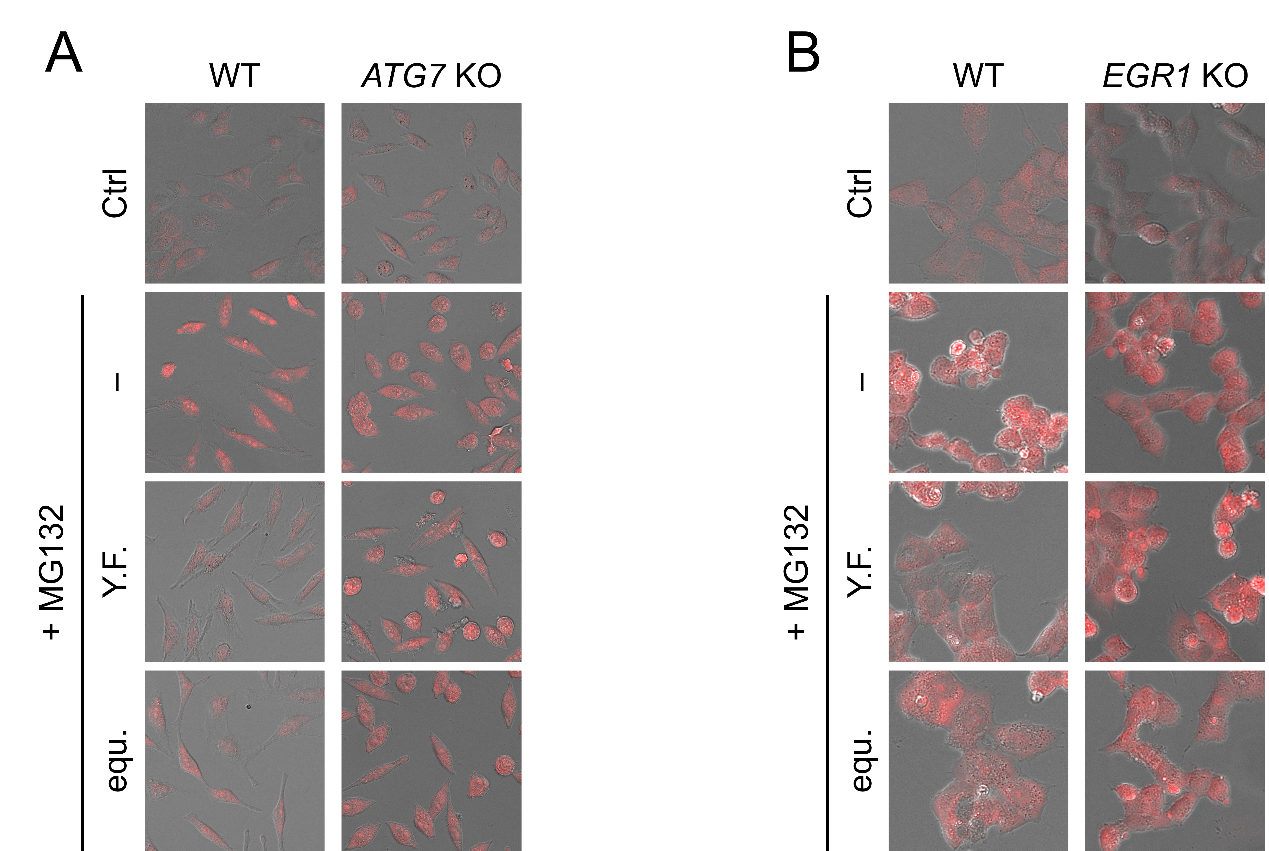


**Supplementary Figure 5. (A)** Cell imaging of Hela-WT and Hela-ATG7 KO cells staining with 10 μM of MitoSOX™RED (Invitrogen) for 1 h. **(B)** HAP11-WT and HAP1-EGR1 KO cells staining with 10 μM of MitoSOX™RED (Invitrogen) for 1 h.

Supplementary Table 1. Primer information used in this study.

| *Homo sapiens* |  | 5′- 3′ |
| --- | --- | --- |
| *GAPDH* | Forward | GAAGGTGAAGGTCGGAGTCA |
|  | Reverse | TGGACTCCACGACGTACTCA |
| *EGR1* | Forward | AGCACCTGACCGCAGAGTCTT |
|  | Reverse | CACTAGGCCACTGACCAAGCT |
| *PIK3C3* | Forward | GCTGTCCTGGAAGACCCAAT |
|  | Reverse | TCAGCCATTCATTCCAGTTCCA |
| *ULK1* | Forward | CAAGATCGCTGACTTCGGCT |
|  | Reverse | CACTGGTAGACGATGGTGCC |
| *ATG4B* | Forward | TCGCTGTGGGGTTTTTCTGT |
|  | Reverse | AGAATCTAGGGACAGGTTCAGGA |
| *ATG7* | Forward | TAGCAGCCCACAGATGGAGTA |
|  | Reverse | TCCCATGCCTCCTTTCTGGT |
| *MAP1LC3B* | Forward | AAGGCGCTTACAGCTCAATG |
|  | Reverse | CTGGGAGGCATAGACCATGT |
| *SQSTM1* | Forward | ACCTTCTGGGCAAGGAGGACGC |
|  | Reverse | CCCCGTCCTCATCGCGGTAGTG |
| Mus musculus |  |  |
| *Actb* | Reverse | CATTGCTGACAGGATGCAGAAGG |
|  | Forward | TGCTGGAAGGTGGACAGTGAGG |
| *Egr1* | Reverse | CCACAACAACAGGGAGACCT |
|  | Forward | ACTGAGTGGCGAAGGCTTTA |
| *Lamp1* | Reverse | ACATCAGCCCAAATGACACA |
|  | Forward | GGCTAGAGCTGGCATTCATC |
| *Sqstm1* | Reverse | GCTCAGGAGGAGACGATGAC |
|  | Forward | AGAAACCCATGGACAGCATC |
| *Map1lc3b* | Reverse | GTCCTGGACAAGACCAAGTTCC |
|  | Forward | CCATTCACCAGGAGGAAGAAGG |
| *Atg7* | Reverse | CCTGTGAGCTTGGATCAAAGGC |
|  | Forward | GAGCAAGGAGACCAGAACAGTG |
